# Transcriptome Profiling Reveals Matrisome Alteration as a Key Feature of Ovarian Cancer Progression

**DOI:** 10.3390/cancers11101513

**Published:** 2019-10-09

**Authors:** Sumegha Mitra, Kartikeya Tiwari, Ram Podicheti, Taruni Pandhiri, Douglas B. Rusch, Andrea Bonetto, Chi Zhang, Anirban K. Mitra

**Affiliations:** 1 Indiana University Melvin and Bren Simon Cancer Center, Indianapolis, IN 46202, USA; abonetto@iu.edu; 2 Department of Obstetrics and Gynecology, Indiana University School of Medicine, Indianapolis, IN 46202, USA; 3 Medical Sciences Program, Indiana University School of Medicine—Bloomington, Bloomington, IN 47405, USA; katiwari@iu.edu (K.T.); tarpandh@iu.edu (T.P.); 4 Center for Genomics and Bioinformatics, Indiana University, Bloomington, IN 47405, USA; mnrusimh@indiana.edu (R.P.); drusch@indiana.edu (D.B.R.); 5 Department of Surgery, Indiana University School of Medicine, Indianapolis, IN 46202, USA; 6 Department of Medical and Molecular Genetics, Indiana University School of Medicine, Indianapolis, IN 46202, USA; czhang87@iu.edu

**Keywords:** ovarian cancer, fallopian tube, primary tumor, metastasis, gene expression, sequencing, tumor microenvironment, matrisome

## Abstract

Background: Ovarian cancer is the most lethal gynecologic malignancy. There is a lack of comprehensive investigation of disease initiation and progression, including gene expression changes during early metastatic colonization. Methods: RNA-sequencing (RNA-seq) was done with matched primary tumors and fallopian tubes (n = 8 pairs) as well as matched metastatic and primary tumors (n = 11 pairs) from ovarian cancer patients. Since these are end point analyses, it was combined with RNA-seq using high-grade serous ovarian cancer cells seeded on an organotypic three-dimensional (3D) culture model of the omentum, mimicking early metastasis. This comprehensive approach revealed key changes in gene expression occurring in ovarian cancer initiation and metastasis, including early metastatic colonization. Results: 2987 genes were significantly deregulated in primary tumors compared to fallopian tubes, 845 genes were differentially expressed in metastasis compared to primary tumors and 304 genes were common to both. An assessment of patient metastasis and 3D omental culture model of early metastatic colonization revealed 144 common genes that were altered during early colonization and remain deregulated even in the fully developed metastasis. Deregulation of the matrisome was a key process in early and late metastasis. Conclusion: These findings will help in understanding the key pathways involved in ovarian cancer progression and eventually targeting those pathways for therapeutic interventions.

## 1. Introduction

Ovarian cancer (OC) is the deadliest gynecologic malignancy and a highly heterogenous disease [1,2,3]. High-grade serous OC (HGSOC) is the most prevalent and aggressive histologic subtype, constituting about 70% of all cases [4]. The other subtypes include endometroid, mucinous and clear cell OC. It was the first cancer to be extensively characterized by The Cancer Genome Atlas (TCGA) study [2], which found tumor protein p53 (TP53) mutations in almost all HGSOC tumors (96%). Among the other mutations, BRCA1 DNA repair associated (BRCA1) and BRCA2 DNA repair associated (BRCA2) were found to be mutated in about 22% of samples, while seven other genes were mutated in 2–6% of tumors [2]. Another defining characteristic was the frequent copy number variations observed in HGSOC tumors. Based on the mRNA expression profiles, OC tumors were subclassified into differentiated, immunoreactive, mesenchymal or proliferative groups. Bowtell et al. had similarly identified six molecular subtypes of OC, which they termed C1–C6 [5]. They found that most high-grade tumors clustered into C1 (high stromal response), C2 (high immune signature), C4 (low stromal response) and C5 (mesenchymal, low immune signature). Together, these two studies provided a molecular overview of the aberrations at the genomic level as well as at the level of gene expression. Such molecular profiling provided a better perspective and opened up opportunities for personalized medicine. Integrated analyses of microarray based gene expression data sets have also resulted in the identification of prognostic signatures for OC [6]. However, these studies were limited to characterizing the primary tumors and did not directly reveal much about the progression and metastasis of OC.

For a more comprehensive understanding of the disease, one has to start from its tissue of origin. For many years, HGSOC was thought to originate from the ovarian surface epithelium. However, strong evidence for fallopian tube (FT) fimbria as the tissue of origin was uncovered when careful study of BRCA mutation carriers and later, HGSOC patients, revealed serous tubal intraepithelial carcinoma (STIC) in their FT fimbria as precursor lesions [7,8,9]. More precisely, a multi-platform genomic study revealed that even in those HGSOC cases, where STICs were not evident in the advanced stage, the potential site of origin remained the distal FT [10]. Even low-grade serous carcinoma of the ovary has now been reported to probably originate from the FT [11]. In recent years, significant progress has been made in our understanding of the genomic landscape of OC carcinogenesis through these sequencing studies. To a lesser extent, groups have also compared metastasis to primary tumors [12,13]. While the study by Grellety et al. was limited to comparing a panel of 429 genes in primary and metastatic tumors, Marchion et al. did a microarray analysis of ovarian surface epithelial cells, pelvic tumors and extrapelvic tumors. Another study based on RNA-seq of primary and metastatic tumors from omentum and bowel focused on phylogenetic analysis to determine the sequence of progression [14]. A study comparing matched primary tumors and omental metastasis for DNA copy number and mRNA expression changes identified the development of an aggressive phenotype with metastasis [15]. A recent report profiling gene expression before and after neoadjuvant chemotherapy showed marked changes in gene expression, including increased drug transport and peroxisomal pathways [16]. However, very few studies have done a comprehensive profiling of OC progression by comparing fallopian tube with primary tumor and metastasis, to identify the key pathways deregulated during carcinogenesis and subsequent progression. For example, a study based on exome sequencing of matched fallopian tube, ovarian tumor and metastasis from an OC patient revealed that transcoelomic metastasis did not result in much accumulation of genetic alterations [17]. In addition, a whole-exome sequencing of matched STICs, invasive fallopian tube carcinoma, ovarian tumors and omental metastasis identified diverse metastatic paths in OC, including STICs formed as a result of metastasis [18].

Due to these facts, a similar comprehensive approach comparing the transcriptome of fallopian tube with primary tumors and metastasis would be beneficial in revealing the oncogenic pathways altered, independent of genetic alterations. Since it has been demonstrated that accumulation of genetic changes is limited during metastasis [17], an RNA-seq based approach will be relevant to understand microenvironment induced gene expression changes. During metastasis, the OC cells encounter a new microenvironment that results in induction of adaptive changes [19]. Signals from the microenvironment have been shown to induce transcription factor and microRNA expression changes, which drive metastatic colonization, by changing the expression of their targets [20,21]. Similarly, interactions with the metastatic microenvironment can induce epigenetic changes such as DNA methylation through the induction of DNA methyl transferase [21]. While using RNA-seq for performing end point analysis of patient tumors is a relevant approach, it still does not provide any information of the intermediate steps and their regulation during metastasis. Since metastasis is a multi-step process, such information about the individual steps will be helpful in understanding and targeting the process that is mainly responsible for poor patient outcomes [22]. In vitro models, that can mimic certain steps of OC metastasis have been increasingly employed to understand the underlying mechanisms regulating these steps [23,24,25]. We have used such an organotypic three-dimensional (3D) culture model of the omentum—a common site of OC metastasis—to study the early regulation of metastatic colonization [20,21]. This model provides an insight into early metastatic colonization—the rate limiting step in metastasis [26]. In the present study, we have used a comprehensive approach of combining the endpoint RNA-seq analysis of OC patients’ normal fallopian tubes, primary ovarian tumors and metastatic tumors with that of the transcriptomic changes occurring during early metastatic colonization, using the organotypic omentum culture model. This study not only reveals the key pathways deregulated during carcinogenesis and metastasis, but also provides a better understanding of the adaptive process induced during metastatic colonization. Such an approach revealed evidences that changes in the matrisome and related pathways are a defining feature of OC progression.

## 2. Results

### 2.1. Study Design

RNA isolated from eight pairs of matched normal FT and primary tumors from OC patients were sequenced to identify the transcriptome changes during carcinogenesis. Similarly, RNA isolated from eleven pairs of matched primary and metastatic tumors were sequenced. Patient characteristics are included in Supplementary Table S1. This was combined with sequencing of RNA from three different HGSOC cells (Kuramochi, OVCAR4 and OVCAR8) seeded on the organotypic 3D culture model of the omentum [20,21] compared to controls, to identify the gene expression changes occurring during early metastatic colonization. Extensive analysis of the RNA-seq data, including differential expression, gene set enrichment, pathway analysis and interaction networks, was performed to identify the genes and pathways altered during carcinogenesis compared to metastasis as well as early metastatic colonization (3D omentum culture model) versus advanced metastasis (patient tumors).

### 2.2. Gene Expression Changes in Normal FT versus Primary Tumors and Primary Tumors versus Metastasis

**Figure 1:**
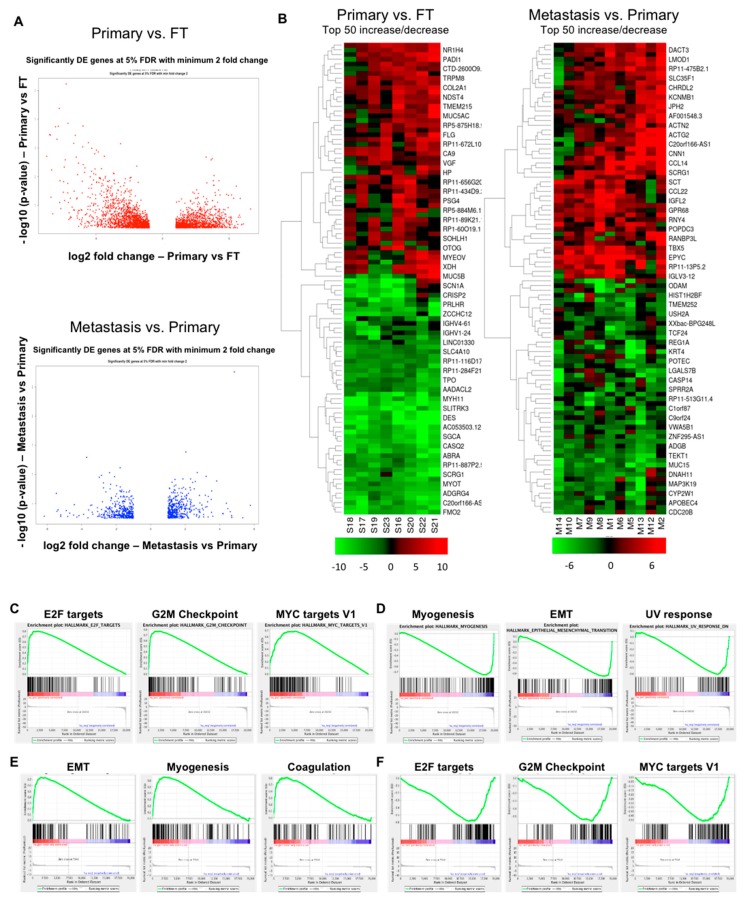
Gene expression analysis of ovarian carcinogenesis and progression. (**A**) Top: Volcano plot representing differentially expressed (DE) genes for primary tumors versus fallopian tube. Bottom: Volcano plot representing differentially expressed genes for metastasis versus primary tumors. The -log10 (*p*-values) plotted against log2 fold change values for all the significantly differentially expressed genes at 5% false discovery rate (FDR) with at least a two-fold change between Primary versus fallopian tube tumors (FT) (top) and Metastasis versus Primary tumors (bottom). The dots on the negative and positive values of X-axis in the top figure represent downregulated and upregulated genes, respectively, in Primary tumors relative to FT. The bottom figure represents the same in metastatic tumors relative to primary tumors. (**B**) Heat map representing top 50 upregulated and downregulated genes (fold change, at least 5% FDR) in primary tumors versus fallopian tube (Left) and in metastasis versus primary tumors (Right). Elevated gene expressions are depicted by increasingly deeper shades of red, while the diminished levels of expression are indicated by deeper shades of green. (**C**) Gene set enrichment analysis (GSEA) in primary tumors versus fallopian tube (positively correlated). (**D**) Primary tumors versus fallopian tube (negatively correlated). (**E**) GSEA in metastasis versus primary tumors (positively correlated). (**F**) Metastasis versus primary tumors (negatively correlated).

A total of 2987 genes were differentially expressed in the primary tumor samples versus FT with a significance level of false discovery rate (FDR) < 5% and absolute fold change ≥ 2 (Figure 1A, Top; Supplementary Figure S1A), whereas 845 genes were differentially expressed in metastasis versus primary tumor samples with a significance level of FDR < 5% and absolute fold change ≥ 2 (Figure 1A, Bottom; Supplementary Figure S1B). Butterfly plots demonstrating the positive and negative correlation of the genes in these two datasets are provided in Supplementary Figure S2. In order to identify the key deregulated genes, the top 50 upregulated and downregulated genes (fold change) were determined for primary tumor versus FT and for metastasis versus primary tumors. Heat maps were generated for these top 50 upregulated and downregulated genes (Figure 1B).

Gene set enrichment analysis (GSEA) was done for the hallmark gene sets to identify the deregulated pathways during OC progression. 15 gene sets were positively correlated with primary tumors versus FT at FDR < 25% while four gene sets were negatively correlated at FDR < 25%. The most significantly correlated gene sets in primary tumors versus FT were E2f transcription factor (E2F) targets (enrichment score (ES) = 0.804), G2M Checkpoint (ES = 0.776) and MYC targets V1 (ES = 0.692) (Figure 1C). Taken together, these results clearly indicate that during the process of carcinogenesis, the tumor acquires a proliferative phenotype where cell cycle regulation is affected along with the activation of oncogenic pathways. Gene sets involving myogenesis (ES = −0.756), epithelial–mesenchymal transition (EMT) (ES = −0.652) and ultraviolet (UV) response (ES = −0.611) were the most significantly negatively correlated in primary tumors versus FT (Figure 1D). EMT transition (ES = 0.741), myogenesis (ES = 0.655) and coagulation (ES = 0.613) are enriched in the metastasis (Figure 1E), highlighting the importance of invasiveness and contractility during the process of metastasis. The E2F targets are important for carcinogenesis but are downregulated during metastasis (ES = −0.586) (Figure 1F). Interestingly, G2M checkpoint (ES = −0.566) and MYC targets V1 (ES = −0.516) are inversely correlated to metastasis (Figure 1F) showing that the primary tumors are more reliant on the proliferative phenotype.

Other significant Hallmark gene sets positively correlating with primary tumors versus FT were mTOR complex 1 (MTORC1) signaling, MYC targets V2, DNA repair and oxidative phosphorylation (Supplementary Figure S3A). Additionally, apical junction, tumor necrosis factor a (TNFA) signaling via nuclear factor kappa B (NFKB), adipogenesis and angiogenesis (Supplementary Figure S3B) were negatively correlated. The metastasis versus primary tumors revealed additional gene sets that positively correlated with metastasis including apical junction, allograft rejection, interleukin 2 (IL2)—signal transducer and activator of transcription 5 (STAT5) signaling and adipogenesis (Supplementary Figure S3C). Similarly, those that were negatively correlated were MTORC1 signaling, protein secretion, androgen response and MYC targets (Supplementary Figure S3D).

**Figure 2:**
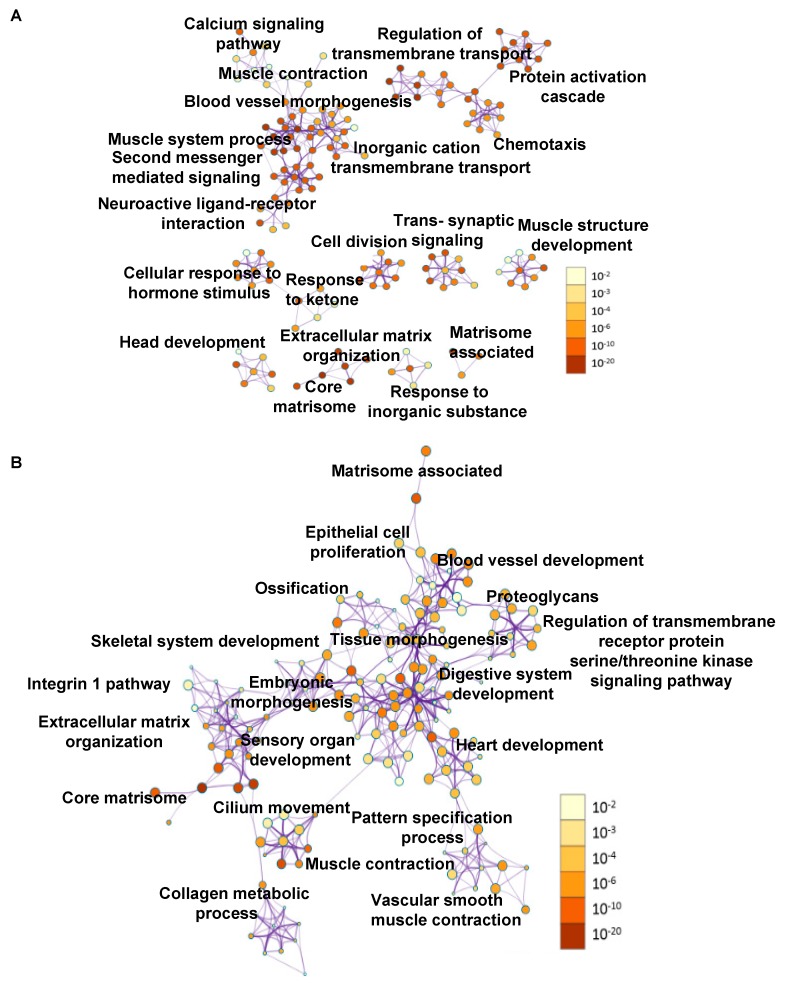
Pathway analysis of deregulated genes. (**A**) Pathway analysis for all deregulated genes in primary tumors versus fallopian tube. The interaction modules are clustered on the basis of functional similarities. (**B**) Pathway analysis for all deregulated genes in metastasis versus primary tumors with interaction modules clustered based on functional similarities. The node color intensity corresponds to the gene enrichment for a particular pathway and the node size correlates with statistical significance.

**Figure 3:**
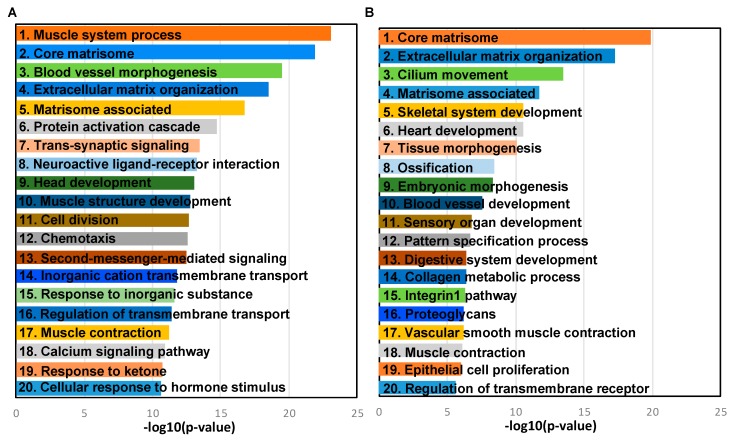
Deregulated pathways. (**A**) Most significantly deregulated pathways in primary tumors versus fallopian tube or (**B**) in metastasis versus primary tumors plotted against −log_10_ (*p*-values).

The primary tumor versus FT and metastasis versus primary tumor data for the top deregulated genes (Figure 1B) were further analyzed for pathways deregulated using Metascape [27] and presented as ontology clusters in Figure 2A,B, respectively. Muscle system process, core matrisome, blood vessel morphogenesis and extracellular matrix (ECM) organization were the most significant pathways in the primary tumors versus FT (Figure 3A). Core matrisome, ECM organization, cilium movement and matrisome associated were the most significant pathways in metastasis versus primary tumors (Figure 3B). Pathways involving matrisome, ECM organization, cellular signaling, development and morphogenesis were common in both of the data sets. In addition, an Ingenuity Pathway Analysis (IPA) was also conducted taking into consideration all the genes differentially expressed at 5% FDR with at least two-fold change in Primary tumors versus FT (Table 1 (panel A); Supplementary Figure S4A). The top canonical pathways included hepatic fibrosis/hepatic stellate cell activation, cAMP mediated signaling, G-protein coupled receptor signaling, calcium signaling and amyotrophic lateral sclerosis signaling. The top disease functions identified were cancer, cellular development, organismal injury and abnormalities (Table 1 (panel B)). The predicted upstream regulators included transforming growth factor beta 1 (TGFB1) (inhibited), vascular endothelial growth factor (VEGF), β-estradiol and erb-b2 receptor tyrosine kinase 2 (ERBB2) (activated) (Table 1 (panel C)). Similarly, IPA analysis for all differentially expressed genes in metastasis versus primary tumors revealed hepatic fibrosis/hepatic stellate cell activation, osteoarthritis pathway, agranulocyte adhesion, and diapedesis and inhibition of matrix metalloproteases as the top canonical pathways (Table 1 (panel D); Supplementary Figure S4B). The top diseases and functions revealed by the analysis were digestive system development and function, embryonic development, and organismal development, which indicated that the process of metastasis mimicked regulatory mechanisms involved in development (Table 1 (panel E)). The key upstream regulators identified were myocardin (MYOCD) (activated), bone morphogenetic protein 4 (BMP4), RUNX family transcription factor 2 (RUNX2) and bone morphogenetic protein 2 (BMP2) (Table 1 (panel F)).

### 2.3. Overlapping Genes in Primary Tumors versus FT and Metastasis versus Primary Tumors

**Figure 4:**
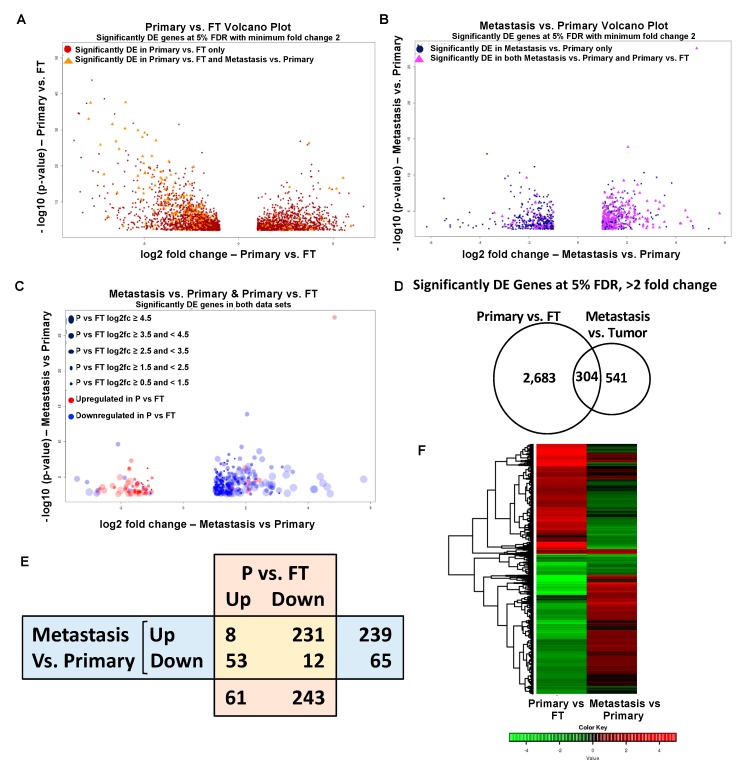
Gene expression analysis in ovarian cancer. (**A**) Volcano plot representing differentially expressed (DE) genes for primary tumors versus fallopian tube (red dots), where the differentially expressed genes common to both primary tumors versus fallopian tube and metastasis versus primary tumors are highlighted (orange triangles). The −log_10_ (*p*-values) plotted against log2 fold change values for all the significantly differentially expressed genes at 5% FDR with at least two-fold change between primary tumors versus FT (**B**) Volcano plot representing differentially expressed genes for metastasis versus primary tumors (blue dots), where the differentially expressed genes common to both primary tumors versus fallopian tube and metastasis versus primary tumors are highlighted (purple triangles). The −log_10_ (*p*-values) plotted against log2 fold change values for all the significantly differentially expressed genes at 5% FDR with at least two-fold change between metastasis versus primary tumors. (**C**) Volcano plot for metastasis versus primary tumors showing only the differentially expressed genes common in both metastasis versus primary tumors and primary tumors versus fallopian tube. Circle size is correlated to the fold change in expression in primary tumors versus fallopian tube. Circles are color coded to depict upregulation (red) or downregulation (blue) in primary tumors versus fallopian tube. (**D**) Venn diagram representing the overlap between primary tumors versus fallopian tube and metastasis versus primary tumors. (**E**) Table representing common upregulated and downregulated genes in ovarian cancer metastasis versus primary tumors and primary tumors (P) versus FT. (**F**) Combined heat map showing the hierarchical clustering based on log2 fold change values of common significantly differentially expressed genes in primary tumors versus fallopian tube and metastasis versus primary tumors.

As the disease progresses from the formation of the primary tumor to the dissemination and development of the metastasis, it is expected to be associated with alterations in gene expression. Our analysis of sequencing data from patient specimens showed that the formation of primary tumors resulted in deregulation of 2987 mRNAs (Supplementary Table S2). The process of metastasis was accompanied by changes in 845 genes (Supplementary Table S3). We next proceeded to visualize the overlap in these two data sets. Figure 4A shows a volcano plot of the 2987 genes deregulated in the Primary tumors versus FT, where the genes that were also found to be differentially expressed in the metastasis versus primary tumors are highlighted as orange triangles. Similarly, the volcano plot of the 845 deregulated genes in metastasis versus primary tumors in Figure 4B highlights the genes that were also differentially expressed in Primary tumors versus FT as purple triangles. We then proceeded to visualize the extent of differential expression and the direction of change of the overlapping genes in both the datasets (Figure 4C). The size of the dots in the volcano plot of the differentially expressed common genes in the metastasis versus primary tumors indicates the extent of differential expression of those genes in Primary tumors versus FT. Similarly, they are also color coded to indicate their upregulation or downregulation in FT versus primary (Figure 4C). Of the 2987 and 845 differentially expressed mRNAs in Primary tumors versus FT and metastasis versus primary tumors, respectively, 304 mRNAs were overlapping (Figure 4D, Supplementary Tables S4 and S5). Interestingly, an analysis of these overlapping genes revealed differences in the direction of change in the two data sets (Figure 4E,F). While a majority of these genes were upregulated in metastasis versus primary tumors, most of them were downregulated in the primary tumors versus FT. This indicated that there were major differences in the processes involved in the initial carcinogenesis and the subsequent dissemination of the disease. The eight upregulated and 12 downregulated genes common to both the comparisons are listed in Table 2 (panels A and B). 231 genes were upregulated in metastasis versus primary tumors but downregulated in primary tumors versus FT. 53 genes were downregulated in metastasis versus primary tumors but upregulated in primary tumor versus FT. The top 10 oppositely regulated genes that are upregulated or downregulated in metastasis versus primary are listed in Table 2 (panels C and D), respectively.

**Figure 5:**
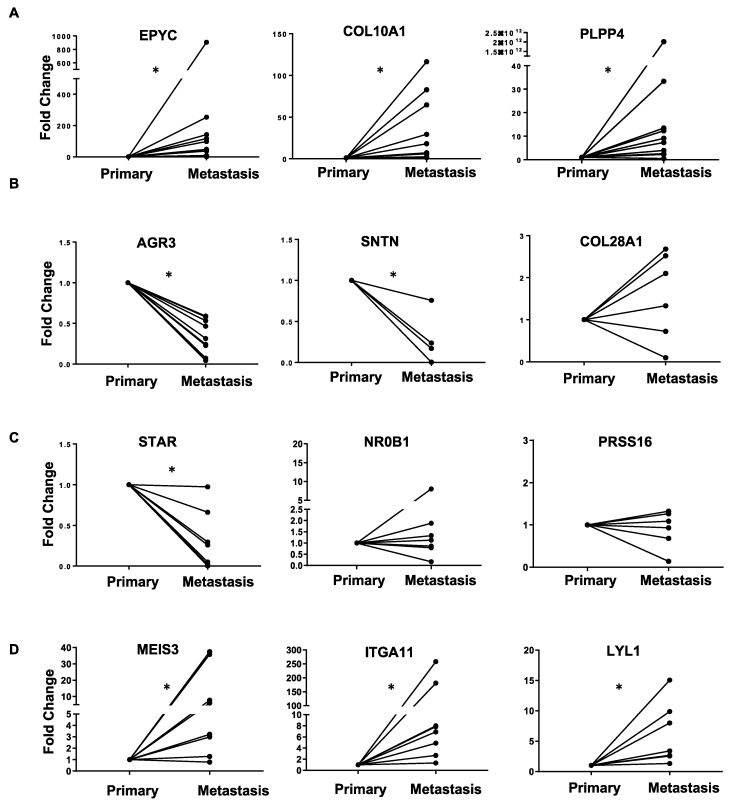
qRT-PCR (quantitative real-time polymerase chain reaction) validation. RNA isolated from matched primary and metastatic ovarian cancer patient tumors were used to perform qRT-PCR for the top deregulated genes in both primary and metastasis. Data represented in the form of before and after plots. (**A**) Genes upregulated in both primary and metastasis. (**B**) Genes downregulated in both primary and metastasis. (**C**) Genes downregulated in metastasis but upregulated in primary. (**D**) Genes upregulated in metastasis but downregulated in primary. * *p* < 0.05 (paired t-test).

**Figure 6:**
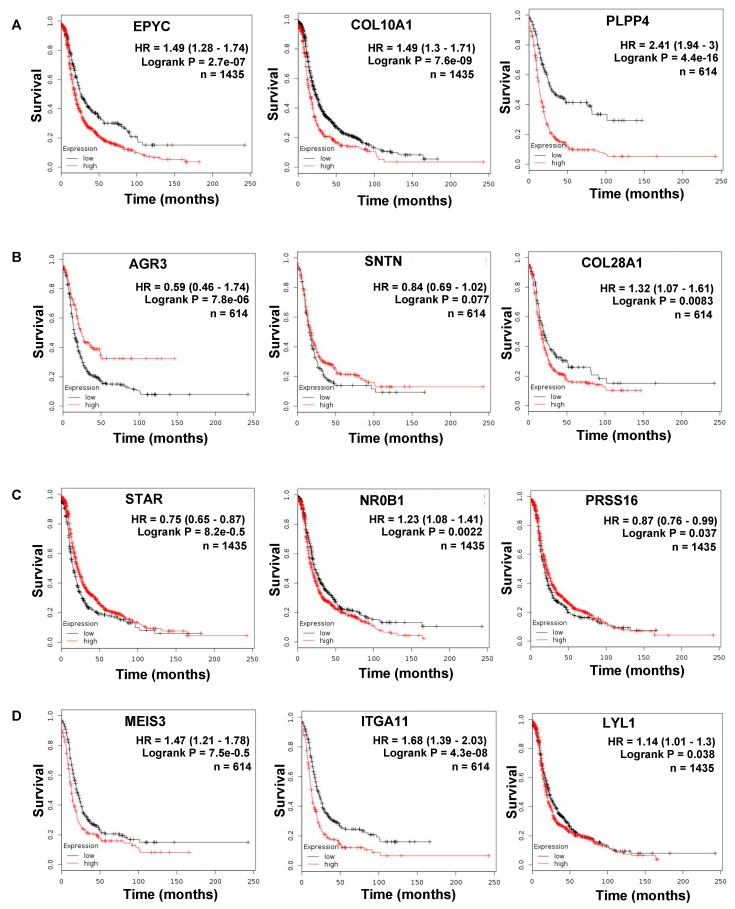
Kaplan-Meier plot for progression free survival analysis for top deregulated genes in both primary tumors and metastasis. (**A**) Genes upregulated in both primary and metastasis. (**B**) Genes downregulated in both primary and metastasis. (**C**) Genes downregulated in metastasis but upregulated in primary. (**D**) Genes upregulated in metastasis but downregulated in primary.

The common upregulated genes in metastasis and primary tumor include epiphycan (EPYC), collagen type X alpha chain 1 (COL10A1), fibronectin type III domain containing 1 (FNDC1) and podocan-like protein I (PODNL1), which are mainly extracellular matrix related genes. These findings further signify the importance of the tumor microenvironment in the progression of OC. The common downregulated genes in metastasis and primary tumor include anterior gradient protein 3 homolog (AGR3) and Sentan cilia apical structure protein (SNTN). Of the 2683 genes that were unique to primary versus FT and 541 genes unique to metastasis versus primary, the top 10 upregulated and downregulated ones are listed in Supplementary Table S6 (panels A–D). Thereafter, we proceeded to confirm the RNA-seq results by qRT-PCR (quantitative real-time polymerase chain reaction), for the top genes in each group of Table 2 in matched ovarian cancer patient primary tumors and metastasis (Figure 5). The top three genes were chosen on the basis of the p-value and the published literature on their role in cancer. We also checked for their effect on ovarian cancer patient prognosis using KM Plotter (Figure 6). Patients were split by median expression of the gene and no exclusion criteria were applied (Supplementary Table S7). *EPYC*, *COL10A1* and *PLPP4,* which were upregulated in both metastasis versus primary and primary versus FT were all confirmed by qRT-PCR. All three genes led to poor patient prognosis when overexpressed (Figure 5A).

Among the genes downregulated in both the datasets, AGR3 and STN were confirmed to be downregulated in metastasis by qRT-PCR, but collagen type XXVIII alpha 1 chain (COL28A1) did not validate this (Figure 5B). Similarly, decreased expression of AGR3 and STN resulted in poor patient prognosis, while COL28A1 had the opposite effect (Figure 6B). Steroidogenic acute regulatory protein (STAR), nuclear receptor subfamily 0 group B member 1 (NR0B1) and serine protease 16 (PRSS16) are downregulated in metastasis versus primary but upregulated in primary versus FT. However, only STAR could be confirmed to be downregulated in metastasis by qRT-PCR (Figure 5C). A decrease in STAR expression resulted in worse prognosis in patients, whereas NR0B1 and PRSS16 had moderate effects (Figure 6C). Meis homeobox 3 (MEIS3), integrin subunit alpha 11 (ITGA11) and LYL1 basic helix-loop-helix family member (LYL1) are increased in metastasis and decreased in primary tumors versus FT. All of them were confirmed to be increased in metastasis by qRT-PCR (Figure 5D). Among them, increased ITGA11 caused the worst prognosis (Figure 6D).

### 2.4. Transcriptome Changes During Early Metastatic Colonization

**Figure 7:**
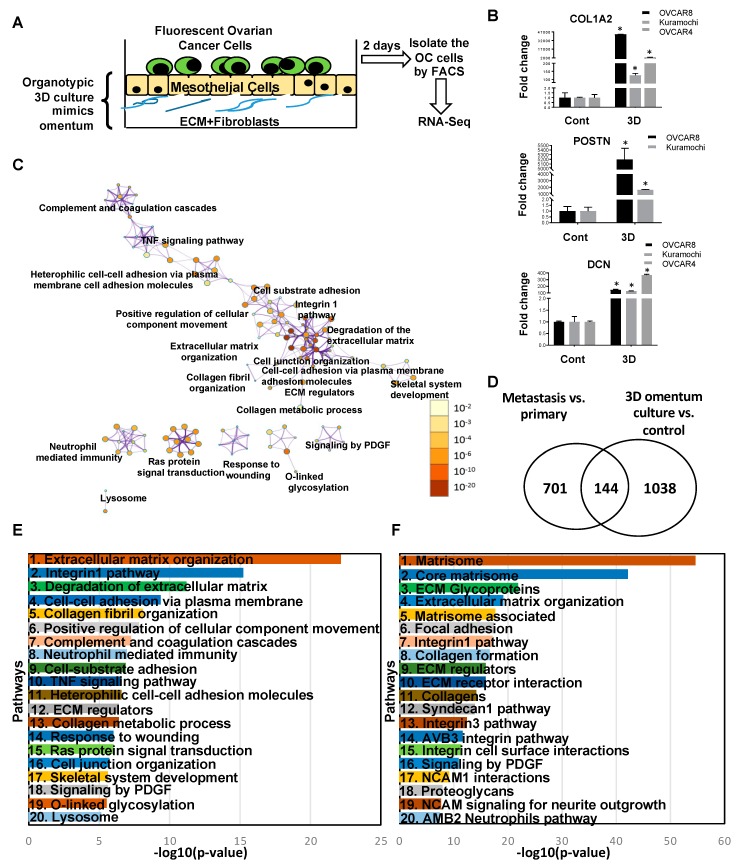
Early and advanced metastatic colonization. (**A**) Schematic of omental three-dimensional (3D) organotypic culture model. Normal omental fibroblasts were mixed with Collagen I, seeded in a culture dish and allowed to attach and polymerize, resembling the basement membrane of the omentum. Omental mesothelial cells were overlaid on it as a confluent monolayer to mimic the mesothelium and the culture was allowed to secrete extracellular matrices (ECMs) and factors for 24 h to form a complex microenvironment. Thereafter, green fluorescent protein (GFP)-labeled high-grade serous ovarian cancer (HGSOC) cells (Kuramochi/OVCAR4/OVCAR8) were seeded on the 3D omentum culture, allowed to grow for 2 days and then isolated by fluorescence activated cell sorting and used for RNA-seq. Three independent experiments were done for each cell. (**B**) The top three differentially expressed genes were validated by qRT-PCR all three cells. * *p* < 0.01, error bars indicate standard deviation for three replicates. (**C**) Network analysis of pathways deregulated in 3D culture versus cell culture where node color intensity corresponds to the gene enrichment for a particular pathway and the node size correlates with statistical significance. The interaction modules are clustered on the basis of functional similarities. (**D**) Venn diagram showing the common deregulated genes in all the 3 HGSOC cells on 3D omentum culture versus control and metastasis versus primary tumors. (**E**) Top 20 deregulated pathways in all the three HGSOC cells seeded on 3D omentum culture versus control plotted against −log_10_ (*p*-values). (**F**) Top 20 common deregulated pathways in all the 3 HGSOC cells seeded on 3D omentum culture versus control and metastasis versus primary tumors plotted against −log_10_ (*p*-values).

The comparison of metastatic tumors with primary tumors of OC patients provides an end-point analysis of the differential gene expression occurring during metastasis. However, metastasis is a multi-step process and the knowledge of the regulation of the individual steps remains limited. The important, rate-limiting step of metastasis is the step of metastatic colonization [28]. At this step, the cancer cells reach the site of metastasis and then have to successfully adapt to the new microenvironment that they encounter [20,21,22]. Such adaptive processes involve productive interactions with the microenvironment and result in changes in gene expression, a process that is very poorly understood. Therefore, in vitro organotypic models, which mimic the microenvironment of the metastatic site, have been used to gain a better understanding of metastatic colonization [20,25,29,30]. Thus, we complimented our end point analysis of patient tumors with the gene expression analysis of HGSOC cells seeded on an organotypic 3D culture model of the human omentum (Figure 7A) [20,21]. Green fluorescent protein (GFP) labeled HGSOC cells Kuramochi, OVACR4 and OVCAR8 were seeded on the organotypic 3D omentum culture model to mimic the early steps of metastatic colonization. This effectively replicates the initial interactions of the OC cells with the metastatic microenvironment and the resulting gene expression changes in the OC cells were analyzed by RNA-seq (Figure 7A, Supplementary Figure S5). The 1182 differentially expressed genes represent the early changes induced by the new microenvironment of the metastatic site (Supplementary Table S8). The top deregulated genes, collagen type I alpha 2 chain (COL1A2), periostin (POSTN) and decorin (DCN) were then validated by qRT-PCR in all HGSOC cells (Figure 7B). POSTN did not amplify in OVCAR4 control, but had an average Cq of 26.8 for OVCAR4 on 3D omentum culture, indicating a marked increase in expression. To identify the pathways involved in early metastatic colonization, a pathway analysis was performed using Metascape [27] for the differentially expressed genes in the HGSOC cells seeded on the organotypic 3D omentum culture model (Figure 7C,E). The most significant pathways included ECM organization, integrin1 pathway, degradation of ECM, cell-cell adhesion via plasma membrane, collagen fibril organization and positive regulation of cellular component movement. Taken together, these indicate that the initial events during metastatic colonization involve interactions of the OC cells with the metastatic microenvironment, remodeling of the ECM and an invasive phenotype. Other pathways, such as the response to wounding, Ras, tumor necrosis factor (TNF) and platelet derived growth factor (PDGF) signaling, indicate early signs of reprogramming of the microenvironment and proliferative growth in close coordination with the microenvironment. The ontology clusters in Figure 7C help visualize the inter-relation between these pathways. While some of these pathways have a significant amount of cross-talk, others appear to constitute independent hubs.

Some of these changes may be transient, while others may be sustained and essential for the metastatic tumor development. Therefore, this dataset was compared with the differentially expressed genes in the end-point analysis of metastasis versus primary tumors to identify those genes essential for both early colonization, as well as for the advanced metastasis (Figure 7D). The 144 genes common to both the data sets represent those that are deregulated early during metastatic colonization, by the interactions with the metastatic microenvironment, and remain deregulated until the end point in the fully developed metastasis (Supplementary Table S9). These genes represent the essential drivers of the process of metastatic colonization and may serve as clinically relevant targets. The pathway analysis was also conducted for the 144 common differentially expressed genes in the metastasis versus primary tumors and HGSOC cells on 3D omentum culture (Figure 7F). The most significant pathways identified were matrisome, core matrisome, ECM glycoproteins, extracellular matrix organization, matrisome associated, focal adhesion and integrin1 pathway. Apart from integrin signaling, PDGF signaling was also among the signaling pathways identified. Therefore, remodeling of the ECM, interactions with the microenvironment and alteration of the matrisome are the key features of the metastatic tumor development. These pathways also indicate the importance of the tumor microenvironment in the metastatic progression, and this needs to be considered for the development of new therapeutic strategies.

## 3. Discussion

In the current study, we did a comprehensive RNA-seq analysis of normal fallopian tubes, primary tumors and metastasis from OC patients, in combination with an organotypic 3D omental culture model for early metastatic colonization. While much information is available on the gene expression profiling of OC primary tumors compared to normal tissue, the information available about metastasis is still limited [31]. Therefore, a comprehensive study of carcinogenesis and progression together, to identify the common and unique signatures, is essential. Moreover, not much is known about the pathways deregulated during early metastatic colonization, the rate-limiting step of metastasis. Here, we combined end point analysis of primary versus metastatic tumors from patients with a 3D culture model of early metastatic colonization, to identify the common pathways deregulated during early colonization that remain relevant in advanced metastasis. Our results indicated that the core matrisome, extracellular matrix components, focal adhesion pathways, integrin mediated pathways and collagen formation pathways were clearly essential for this process.

It has been well documented that the genes coding for ECM components undergo deregulation in tumor progression [32,33,34,35]. Moreover, altered ECMs can drive tumor progression [36,37]. Tumorigenic properties of cancer cells such as migration and proliferation are largely influenced by the ECM composition and ultrastructure. Differential regulation of ECM components has been observed in the early and late metastasis stages, which further signifies the contribution of ECM in tumor progression [38]. Tumor extracellular matrix is contributed by both tumor cells and the stromal cells [38]. It has been demonstrated in OC that the dysregulation of ECM components and stiffness impacts the OC progression [39].

Collagen acts as a scaffold in tumor ECM and the synthesis and degradation of collagen largely impacts the metastatic attributes of cancer cells. Collagen is found in abundance in the ECM [40,41]. The deposition of collagen I, II and III is increased during cancer invasion and migration [42,43]. Rapid changes involving collagen deposition have also been observed in breast cancer. Collagen type I, II and IV have been utilized as potential biomarkers in colorectal cancer since degraded collagen fragments are released in the circulation [44]. Collagen VI has been found to be overexpressed in OC [45], while Collagen XI is greatly increased in the OC metastasis [46,47,48]. A comprehensive analysis of OC omental metastasis, including gene expression, matrisome, extracellular matrix organization, biomechanical properties, cytokine/chemokine levels and cellular profiles revealed matrisome changes are a defining feature and could be correlated with prognosis [49]. These findings also showed the close correlation between RNA-seq data with matrisome focused proteomics increase in ECM glycoproteins, such as fibronectin and fibrinogen, along with proteoglycans and secreted factors defined metastatic progression [49]. Our results indicate that core matrisome changes and ECM reorganization are key features of initial carcinogenesis and subsequent metastasis, including early metastasis (Figure 2 and Figure 5). The remodeling of the tumor microenvironment was found to be an essential feature, consistent throughout the process of tumor initiation and progression.

Focal adhesions mediate the contact between cell and extracellular matrix, helping anchor the cell to the substratum and promote motility, playing a major role in metastasis [50,51,52,53]. Factors mediating or affecting the focal adhesions are usually upregulated in the tumors and this is also reflected in our sequencing data (Figure 7F). The anchorage to ECMs is mediated by specific heterodimeric membrane glycoproteins—integrins, which interact with various ECM components such as fibronectin, collagen, vitronectin and laminin. Thus, elevated levels of specific integrins and their associated proteins are observed in the metastasis [54,55,56]. Tumor progression is also partly mediated by the vascular integrins, which are expressed in tumor vasculature [57,58].

Primary tumors differentially expressed 2987 genes as compared to the normal fallopian tubes. However, the process of metastasis resulted in changes in the expression of only 845 genes. Of these, 304 genes overlapped with those deregulated in primary tumors versus fallopian tubes. An interesting observation in our study was that these 304 common deregulated genes in primary tumors versus normal fallopian tube and metastasis versus primary tumors were not necessarily altered in the same direction (Figure 4). This implied that certain changes necessary for the initial tumorigenesis were not essential for subsequent metastasis. Therefore, the process of cancer dissemination not only needed a new set of genes but the expression of certain genes that were overexpressed/repressed in the primary tumors had to be reversed. We went on to validate the top genes deregulated in similar and/or opposite directions by qRT-PCR in matched patient primary tumors and metastasis. Among the 12 genes tested, nine of them were validated by qRT-PCR (Figure 5). Among them, increased expression of EPYC, COL10A1, PLPP4, MEIS3 and ITGA11 appeared clinically relevant as markers of poor prognosis (Hazard ratio 1.49, 1.49, 2.41, 1.47 and 1.68, respectively; Figure 6). On the other hand, increased expression of AGR3 was a positive prognosis marker (Figure 6B). Of note, the datasets used for patient prognosis are mostly derived from primary tumor samples.

A key factor to consider is the different microenvironments of the primary tumor and the site of metastasis. Since gene expression changes can be regulated by the signals emanating from the microenvironment, this could be an important reason for the differences in metastasis. Such changes may not be reflected by genetic mutations in the cancer cells. The metastasizing cancer cells have to adapt to the new microenvironment and this is stimulated by the productive cross-talk with the microenvironment [28,59]. Our previous studies have shown that regulators of gene expression, such as microRNAs and transcription factors, are induced or repressed as a result of such reciprocal signaling [20,21]. Similarly, the cancer cells are also capable to induce mesothelial to mesenchymal transition in the mesothelial cells covering the omentum [24,60]. Our findings with the 3D omental culture model and comparison of patient primary and metastasis tumor samples, revealed that many of such initial adaptive changes continue to remain relevant in the advanced metastasis. These changes include multiple pathways involved in the interactions between the tumor cells with their microenvironment (Figure 7F). These results support the recent observations made upon deconstructing omental metastasis [49], while demonstrating that many of these processes start really early during the process of metastatic colonization. These pathways are, therefore, needed for early colonization and remain essential for the subsequent progression into the advanced metastasis.

## 4. Materials and Methods

### 4.1. Reagents

Dulbecco’s Modified Eagle Medium (DMEM), minimal essential medium vitamins, minimal essential medium nonessential amino acids, Trypsin and Penicillin–Streptomycin were obtained from Media Tech (Manassas, VA, USA).

### 4.2. Patient Samples

Matched pairs of fresh frozen primary tumors and normal fallopian tubes from eight OC patients were obtained from the Indiana University Simon Cancer Center Tissue Bank. Similarly, matched pairs of fresh frozen primary tumors and metastasis from 11 OC patients were also obtained from the Indiana University Simon Cancer Center Tissue Bank. RNA was isolated from these tissues using miRNeasy kit (Qiagen, Germantown, MD; Cat# 217004) following the manufacturer’s protocol and submitted to the Center for Genomics and Bioinformatics core facility, Indiana University, Bloomington for library preparation and sequencing. The study was approved by the Institutional Regulatory Board of Indiana University (protocol numbers 1106005767 and 1606070934). Patient written consent was obtained and tissues were collected by Indiana University Simon Cancer Center Tissue Bank. Only deidentified patient specimens were provided by the tissue bank.

### 4.3. Cell Lines

Human HGSOC cell lines OVCAR8 were acquired from Ernst Lengyel at the University of Chicago and OVCAR4 was from Joanna Burdette, University of Illinois at Chicago. Kuramochi was procured from Japanese Collection of Research Bioresources. The cell lines used were genetically validated and tested to be mycoplasma free using respective services from Idexx BioResearch (Columbia, MO). The genetic validation was done using the CellCheck 16 (16 Marker STR Profile and Inter-species Contamination Test) and mycoplasma testing was done using Stat-Myco.

### 4.4. D Omental Culture and RNA Isolation

Total RNA was isolated from a panel of HGSOC cell lines (Kuramochi, OVCAR4 and OVCAR8) grown in 3D omental cultures as described previously [20]. Three independent experiments per HGSOC cell line were conducted. Briefly, human primary mesothelial cells (HPMCs) and normal omental fibroblasts (NOFs) were isolated from omentum of women donors undergoing surgery at Indiana University Health Bloomington Hospital. The NOFs and HPMCs were grown as primary cultures for 2–3 passages and used for the experiments. The NOFs (3.6 × 10^5^ cells) were mixed with 91 μg Collagen I (BD Biosciences, San Jose, CA;Cat# 354236) and seeded in a 10 cm dish and allowed to attach for 4 h. Thereafter, 3.6 × 10^6^ HPMCs were overlaid on them to form a confluent monolayer. This 3D omentum culture mimicking the surface layers of the omentum was allowed to grow for 24 h so that the cells could secrete ECMs and other factors to form a complex microenvironment. GFP labelled OC cells were then seeded on the 3D omentum culture to mimic the initial steps of colonization and allowed to grow for 48 h. The cancer cells were then isolated by fluorescence activated cell sorting and compared to control cancer cells directly seeded on 10 cm tissue culture dishes and sham sorted. Total RNA was isolated using miRNeasy kit (Qiagen, Germantown, MD; Cat# 217004) and submitted for RNA-seq. Only those genes that were significantly differentially expressed (< 5% FDR and absolute fold change ≥ 2) in all three HGSOC cells were considered for further analysis. We chose to use Kuramochi, OVCAR4 and OVCAR8 as they have been reported as ‘likely/possibly’ HGSOC cell lines [61]. Moreover, among the HGSOC cell lines, we have found them to be more suitable for functional assays and OVCAR8 also grows effectively in mouse xenografts [62,63]. Therefore, the same cells would be suitable for extending these studies in the future.

### 4.5. RNA Sequencing

Library preparation and next generation RNA sequencing was carried out at the Center for Genomics and Bioinformatics core facility, Indiana University, Bloomington. The library preparation was done using TruSeq Stranded mRNA HT Sample Prep kit (Illumina, San Diego, CA; cat#RS-122-2103) according to the manufacturer’s protocol and 8-nucleotide barcodes were added for multiplexing. The barcoded libraries were cleaned by bead cut with AMPure XP beads (Beckman Coulter, Atlanta, GA; cat#A63882), verified using Qubit3 fluorometer (ThermoFisher Scientific, Waltham, MA) and 2200 TapeStation bioanalyzer (Agilent Technologies, Santa Clara, CA), and then pooled. The pool was sequenced on NextSeq 500 (Illumina) with NextSeq75 High Output v2 kit (Illumina, cat#FC-404-2005).

### 4.6. Analysis of Sequencing Data

Reads were adapter trimmed and quality filtered using Trimmomatic ver. 0.33 [64], with the cutoff threshold for average base quality score set at 20 over a sliding window of three bases. Reads shorter than 20 bases post-trimming were excluded. Cleaned reads mapped to human genome GRCh38 with gencode v25 annotation using Tophat2 ver 2.1.0 [65]. The number of reads mapped to annotated genes was counted using htseq-counts ver. 0.5.4p1 [66]. Significantly differentially expressed genes at 5% FDR with at least two-fold change were called using DESeq2 ver. 1.12.3 [67]. Categories based on overlaps among significantly differentially expressed genes between the two experiments (primary tumors versus FT and metastasis versus primary tumors) were analyzed using IPA (QIAGEN Inc., https://www.qiagenbioinformatics.com/products/ingenuity-pathway-analysis) and Metascape (metascape.org). Metascape is a web-based tool that has been under development since 2014 [27]. It enables multiple computational analysis of large scale data integrating gene annotation, membership analysis and has multi-gene-list meta-analysis capabilities. It allows the leveraging of 40 independent databases for studying functional enrichment, interactomes and gene annotation. Using Metascape, we ranked the deregulated pathways on the basis of –log_10_ (*p*-values). Interaction modules were also clustered on the basis of functional similarity by Metascape.

Gene set enrichment analysis was conducted using GSEA Desktop ver. 3.0, build 0160 [68]. Hallmark gene sets from Molecular Signatures Database ver. MSigDB 6.1 were used. GSEArot algorithm [69] was used to compute significance of enrichment for each of the hallmark gene sets, taking into consideration that the primary tumors and the FT as well as the metastatic and primary tumors were matched specimens from individual patients.

Heat maps were generated using the heatmap.2 function from R programming tools (gplots).

All RNA-seq data have been made available to the public (GEO accession numbers GSE137237, GSE137238, GSE137239).

## 5. Conclusions

Taken together, our RNA-seq data has generated a wealth of information in the form of crucial pathways and targets which can be further explored to unravel the molecular mechanisms regulating the OC metastasis. Overall, the results of this study emphasize the need to further explore the tumor microenvironment and extracellular matrix components in the progression of OC metastasis. OC patients typically present with the whole spectrum of metastasis ranging from early lesions to advanced metastasis. Targeting key components of the pathways identified in Figure 7F, or their regulators, could be an effective approach to treat the whole spectrum of metastasis.

## Figures and Tables

**Table 1:** Ingenuity pathway analysis (IPA) of differentially regulated genes in ovarian cancer. (panel **A**) Top 20 canonical pathways, (panel **B**) Top disease functions and (panel **C**) predicted upstream regulators in primary tumors versus FT. (panel **D**) Top 20 canonical pathways, (panel **E**) top disease functions and (panel **F**) predicted upstream regulators in metastasis versus primary tumors.

**A**	**Ingenuity Canonical Pathways: FT vs. Primary Tumors**		**−log(*p*-Value)**	**D**	**Ingenuity Canonical Pathways: Metastasis vs. Primary Tumors**		**−log(*p*-Value)**
1	Hepatic Fibrosis / Hepatic Stellate Cell Activation		10.2	1	Hepatic Fibrosis / Hepatic Stellate Cell Activation		6.58
2	cAMP-mediated signaling		10.1	2	Osteoarthritis Pathway		5.15
3	G-Protein Coupled Receptor Signaling		8.16	3	Agranulocyte Adhesion and Diapedesis		4.12
4	Calcium Signaling		6.94	4	Inhibition of Matrix Metalloproteases		2.98
5	Amyotrophic Lateral Sclerosis Signaling		6.08	5	Bladder Cancer Signaling		2.86
6	eNOS Signaling		5.8	6	Atherosclerosis Signaling		2.27
7	MSP-RON Signaling Pathway		5.77	7	Granulocyte Adhesion and Diapedesis		2.07
8	Cellular Effects of Sildenafil (Viagra)		5.34	8	Neuroprotective Role of THOP1 in Alzheimer’s Disease		1.99
9	Intrinsic Prothrombin Activation Pathway		5.31	9	Eicosanoid Signaling		1.8
10	Breast Cancer Regulation by Stathmin1		5.27	10	ILK Signaling		1.76
**B**	**Top Diseases and Functions: FT vs. Primary Tumors**			**E**	**Top Diseases and Functions: Metastasis vs. Primary Tumors**		
1	Cancer, Cellular Development, Organismal Injury and Abnormalities			1	Digestive System Development and Function, Embryonic Development, Organismal Development		
2	Nervous System Development and Function, Cell Death and Survival, Tissue Morphology			2	Cell-mediated Immune Response, Cellular Movement, Hematological System Development and Function		
3	Cell Morphology, Cellular Assembly and Organization, Cellular Function and Maintenance			3	Cardiovascular Disease, Hereditary Disorder, Organismal Injury and Abnormalities		
4	Cell Signaling, Neurological Disease, Organismal Injury and Abnormalities			4	Cardiac Arrythmia, Cardiovascular Disease, Hereditary Disorder		
5	Cellular Development, Cellular Growth and Proliferation, Hematological System Development and Function			5	Skeletal and Muscular System Development and Function, Gastrointestinal Disease, Organismal Injury and Abnormalities		
6	Cancer, Organismal Injury and Abnormalities, Carbohydrate Metabolism			6	Cardiovascular Disease, Cell-To-Cell Signaling and Interaction, Drug Metabolism		
7	Cell Signaling, Carbohydrate Metabolism, Small Molecule Biochemistry			7	Digestive System Development and Function, Connective Tissue Development and Function, Connective Tissue Disorders		
8	Molecular Transport, Connective Tissue Disorders, Developmental Disorder			8	Developmental Disorder, Hereditary Disorder, Ophthalmic Disease		
9	Skeletal and Muscular System Development and Function, Skeletal and Muscular Disorders, Hereditary Disorder			9	Dermatological Diseases and Conditions, Inflammatory Disease, Organismal Injury and Abnormalities		
10	Cell-To-Cell Signaling and Interaction, Cellular Assembly and Organization, Nervous System Development and Function			10	Endocrine System Disorders, Organ Morphology, Organismal Injury and Abnormalities		
**C**	**Upstream Regulator**	**Molecule Type**	***p*-Value of Overlap**	**F**	**Upstream Regulator**	**Molecule Type**	***p*-Value of Overlap**
1	TGFB1	growth factor	1.06 × 10^−38^	1	MYOCD	transcription regulator	1.08 × 10^−13^
2	Vegf	Group	1.48 × 10^−33^	2	BMP4	growth factor	2.57 × 10^−10^
3	beta-estradiol	chemical—endogenous mammalian	3.47 × 10^−29^	3	RUNX2	transcription regulator	6.84 × 10^−10^
4	ERBB2	Kinase	2.48 × 10^−28^	4	BMP2	growth factor	1.43 × 10^−8^
5	dexamethasone	chemical drug	3.2 × 10^−26^	5	miR-199a-5p (and other miRNAs w/seed CCAGUGU)	mature microrna	2.25 × 10^−8^
6	HGF	growth factor	7.39 × 10^−25^	6	U0126	chemical—kinase inhibitor	2.42 × 10^−8^
7	FGF2	growth factor	6.31 × 10^−24^	7	TGFB3	growth factor	2.73 × 10^−8^
8	progesterone	chemical—endogenous mammalian	1.43 × 10^−23^	8	TGFB1	growth factor	3.72 × 10^−8^
9	TNF	cytokine	3.83 × 10^−22^	9	GNA13	enzyme	1.29 × 10^−7^
10	IL6	cytokine	1.87 × 10^−21^	10	HAND2	transcription regulator	2.1 × 10^−7^

**Table 2:** Common genes in metastasis versus primary tumors and primary tumors versus FT.

**(A). Common Upregulated Genes in Ovarian Cancer Metastasis versus Primary and Primary versus FT**			**(B). Common Downregulated Genes in Ovarian Cancer Metastasis versus Primary and Primary versus FT**		
**Gene Name**	**log2 Fold Change**	***p*-Value**	**Gene Name**	**log2 Fold Change**	***p*-Value**
*EPYC*	4.84	2.78 × 10^−28^	AGR3	−2.09	2.25 × 10^−10^
*COL10A1*	1.98	5.19 × 10^−7^	SNTN	−3.09	4.80 × 10^−5^
*PLPP4*	2.06	5.95 × 10^−5^	ANKUB1	−3.41	1.64 × 10^−5^
*RP11-13P5.2*	2.44	2.46 × 10^−5^	COL28A1	−1.29	0.000236
*PODNL1*	1.44	0.002166	DCDC2B	−3.01	0.001219
*CILP2*	1.54	0.000325	ADGB	−2.75	0.000667
*FNDC1*	2.32	3.26 × 10^−5^	CFAP52	−1.88	1.19 × 10^−5^
*SLC35D3*	2.09	0.000901	RP11-356K23.1	−2.77	0.001993
			WDR38	−1.49	8.60 × 10^−5^
			CFAP221	−1.19	0.000604
			NWD1	−1.72	0.000766
			FAM166B	−1.07	0.001892
**(C). Top 10 Opposite: Upregulated Genes in Metastasis versus Primary and Downregulated in Primary versus FT**			**(D). Top 10 Opposite: Downregulated Genes in Metastasis versus Primary and Upregulated in Primary versus FT**		
**Gene Name**	**log2 Fold Change**	***p*-Value**	**Gene Name**	**log2 Fold Change**	***p*-Value**
*MEIS3*	2.04	1.29 × 10^−14^	ATP6V1C2	−1.73	6.83 × 10^−7^
*ITGA11*	2.19	1.58 × 10^−7^	STAR	−1.67	4.38 × 10^−8^
*RP1-79C4.4*	1.10	2.54 × 10^−8^	NR0B1	−1.84	5.01 × 10^−4^
*LYL1*	1.42	9.63 × 10^−7^	KCNG3	−1.58	9.16 × 10^−4^
*ASPA*	1.49	3.70 × 10^−5^	PRSS16	−1.14	3.02 × 10^−5^
*AOC3*	1.49	9.69 × 10^−6^	FAM167A	−1.45	6.31 × 10^−5^
*GIMAP8*	1.41	4.79 × 10^−8^	C3orf67	−1.30	1.00 × 10^−4^
*LRRN4CL*	1.23	5.73 × 10^−5^	JPH1	−1.62	1.85 × 10^−4^
*IGHV1-24*	1.99	5.70 × 10^−4^	HOOK1	−1.56	1.93 × 10^−5^
*CCL14*	2.85	7.45 × 10^−6^	ESM1	−1.78	5.89 × 10^−5^

## Supplementary Materials

The following are available online at https://www.mdpi.com/2072-6694/11/10/1513/s1, Figure S1: Heat maps representing the deregulated genes in ovarian cancer, Figure S2: Butterfly plots, Figure S3: Gene set enrichment analysis (GSEA) for the Hallmark gene set in ovarian cancer, Figure S4: Ingenuity pathway analysis (IPA) of differentially regulated genes in ovarian cancer, Figure S5: Heat map representing differentially expressed genes in all 3 HGSOC cells (Kuramochi/OVCAR4/OVCAR8) seeded on the 3D omentum culture versus control. Table S1: Ovarian cancer patient characteristics and specimen information. Table S2: Differentially expresses genes in primary tumors versus FT. Table S3: Differentially expresses genes in metastasis versus primary tumors. Table S4: 304 differentially expressed genes in primary tumors versus FT that are also deregulated in metastasis versus primary tumors. Table S5: 304 differentially expressed genes in metastasis versus primary tumors that are also deregulated in primary tumors versus FT. Table S6: Common genes in metastasis vs. primary tumors and primary tumors vs. FT. Table S7: KM plotter settings for progression free survival analysis (PFS). Table S8: Differentially expressed genes in Kuramochi, OVCAR4 and OVCAR8 cells seeded on 3D omentum culture. Table S9: The 144 differentially expressed genes that are commonly deregulated in 3D omentum culture and in metastasis versus primary tumors.

## References

[1] Torre L.A., Trabert B., DeSantis C.E., Miller K.D., Samimi G., Runowicz C.D., Gaudet M.M., Jemal A., Siegel R.L. (2018). Ovarian Cancer Statistics, 2018. CA A Cancer J. Clin..

[2] (2011). Integrated Genomic Analyses of Ovarian Carcinoma. Nature.

[3] Vaughan S., Coward J.I., Bast R.C., Berchuck A., Berek J.S., Brenton J.D., Coukos G., Crum C.C., Drapkin R., Etemadmoghadam D. (2011). Rethinking Ovarian Cancer: Recommendations for Improving Outcomes. Nat. Rev. Cancer.

[4] Koonings P.P., Campbell K., Mishell D.R., Grimes D.A. (1989). Relative Frequency of Primary Ovarian Neoplasms: A 10-Year Review. Obstet. Gynecol..

[5] Tothill R.W., Tinker A.V., George J., Brown R., Fox S.B., Lade S., Johnson D.S., Trivett M.K., Etemadmoghadam D., Locandro B. (2008). Novel Molecular Subtypes of Serous and Endometrioid Ovarian Cancer Linked to Clinical Outcome. Clin. Cancer Res..

[6] Denkert C., Budczies J., Darb-Esfahani S., Gyorffy B., Sehouli J., Konsgen D., Zeillinger R., Weichert W., Noske A., Buckendahl A.C. (2009). A Prognostic Gene Expression Index in Ovarian Cancer—Validation across Different Independent Data Sets. J. Pathol..

[7] Piek J.M., van Diest P.J., Zweemer R.P., Jansen J.W., Poort–Keesom R.J., Menko F.H., Gille J.J., Jongsma A.P., Pals G., Kenemans P. (2001). Dysplastic Changes in Prophylactically Removed Fallopian Tubes of Women Predisposed to Developing Ovarian Cancer. J. Pathol..

[8] Callahan M.J., Crum C.P., Medeiros F., Kindelberger D.W., Elvin J.A., Garber J.E., Feltmate C.M., Berkowitz R.S., Muto M.G. (2007). Primary Fallopian Tube Malignancies in BRCA-Positive Women Undergoing Surgery for Ovarian Cancer Risk Reduction. J. Clin. Oncol..

[9] Kindelberger D.W., Lee Y., Miron A., Hirsch M.S., Feltmate C., Medeiros F., Callahan M.J., Garner E.O., Gordon R.W., Birch C. (2007). Intraepithelial Carcinoma of the Fimbria and Pelvic Serous Carcinoma: Evidence for a Causal Relationship. Am. J. Surg. Pathol..

[10] Ducie J., Dao F., Considine M., Olvera N., Shaw P.A., Kurman R.J., Shih I.M., Soslow R.A., Cope L., Levine D.A. (2017). Molecular Analysis of High-Grade Serous Ovarian Carcinoma With and Without Associated Serous Tubal Intra-Epithelial Carcinoma. Nat. Commun..

[11] Li J., Abushahin N., Pang S., Xiang L., Chambers S.K., Fadare O., Kong B., Zheng W. (2011). Tubal Origin of ‘Ovarian’ Low-Grade Serous Carcinoma. Mod. Pathol..

[12] Grellety T., Lucchesi C., Hostein I., Auzanneau C., Khalifa E., Soubeyran I., Italiano A. (2017). High-depth Sequencing of Paired Primary and Metastatic Tumours: Implications for Personalised Medicine. Eur. J. Cancer (Oxford, England: 1990).

[13] Marchion D.C., Xiong Y., Chon H.S., Al Sawah E., Bou Zgheib N., Ramirez I.J., Abbasi F., Stickles X.B., Judson P.L., Hakam A. (2013). Gene Expression Data Reveal Common Pathways that Characterize the Unifocal Nature of Ovarian Cancer. Am. J. Obstet. Gynecol..

[14] Chien J., Neums L., Powell A., Torres M., Kalli K.R., Multinu F., Shridhar V., Mariani A. (2018). Genetic Evidence for Early Peritoneal Spreading in Pelvic High-Grade Serous Cancer. Front. Oncol..

[15] Brodsky A.S., Fischer A., Miller D.H., Vang S., MacLaughlan S., Wu H.T., Yu J., Steinhoff M., Collins C., Smith P.J. (2014). Expression Profiling of Primary and Metastatic Ovarian Tumors Reveals Differences Indicative of Aggressive Disease. PLoS ONE.

[16] Glasgow M.A., Argenta P., Abrahante J.E., Shetty M., Talukdar S., Croonquist P.A., Khalifa M.A., Starr T.K. (2019). Biological Insights into Chemotherapy Resistance in Ovarian Cancer. Int. J. Mol. Sci..

[17] Lee J.Y., Yoon J.K., Kim B., Kim S., Kim M.A., Lim H., Bang D., Song Y.S. (2015). Tumor Evolution and Intratumor Heterogeneity of An Epithelial Ovarian Cancer Investigated Using Next-Generation Sequencing. BMC Cancer.

[18] Eckert M.A., Pan S., Hernandez K.M., Loth R.M., Andrade J., Volchenboum S.L., Faber P., Montag A., Lastra R., Peter M.E. (2016). Genomics of Ovarian Cancer Progression Reveals Diverse Metastatic Trajectories Including Intraepithelial Metastasis to the Fallopian Tube. Cancer Discov..

[19] Bowtell D.D., Bohm S., Ahmed A.A., Aspuria P.J., Bast R.C., Beral V., Berek J.S., Birrer M.J., Blagden S., Bookman M.A. (2015). Rethinking Ovarian Cancer II: Reducing Mortality from High-Grade Serous Ovarian Cancer. Nat. Rev. Cancer.

[20] Tomar S., Plotnik J.P., Haley J., Scantland J., Dasari S., Sheikh Z., Emerson R., Lenz D., Hollenhorst P.C., Mitra A.K. (2018). ETS1 Induction by the Microenvironment Promotes Ovarian Cancer Metastasis Through Focal Adhesion Kinase. Cancer Lett..

[21] Mitra A.K., Chiang C.Y., Tiwari P., Tomar S., Watters K.M., Peter M.E., Lengyel E. (2015). Microenvironment-Induced Downregulation of miR-193b Drives Ovarian Cancer Metastasis. Oncogene.

[22] Mitra A.K. (2016). Ovarian Cancer Metastasis: A Unique Mechanism of Dissemination.

[23] Watters K.M., Bajwa P., Kenny H.A. (2018). Organotypic 3D Models of the Ovarian Cancer Tumor Microenvironment. Cancers.

[24] Kenny H.A., Chiang C.Y., White E.A., Schryver E.M., Habis M., Romero I.L., Ladanyi A., Penicka C.V., George J., Matlin K. (2014). Mesothelial Cells Promote Early Ovarian Cancer Metastasis Through Fibronectin Secretion. J. Clin. Investig..

[25] Iwanicki M.P., Davidowitz R.A., Ng M.R., Besser A., Muranen T., Merritt M., Danuser G., Ince T.A., Brugge J.S. (2011). Ovarian Cancer Spheroids Use Myosin-Generated Force to Clear the Mesothelium. Cancer Discov..

[26] Chaffer C.L., Weinberg R.A. (2011). A Perspective on Cancer Cell Metastasis. Science (New York, N.Y.).

[27] Zhou Y., Zhou B., Pache L., Chang M., Khodabakhshi A.H., Tanaseichuk O., Benner C., Chanda S.K. (2019). Metascape Provides a Biologist-Oriented Resource for the Analysis of Systems-Level Datasets. Nat. Commun..

[28] Chambers A.F., Groom A.C., MacDonald I.C. (2002). Dissemination and Growth of Cancer Cells in Metastatic Sites. Nat. Rev. Cancer.

[29] Peters P.N., Schryver E.M., Lengyel E., Kenny H. (2015). Modeling the Early Steps of Ovarian Cancer Dissemination in an Organotypic Culture of the Human Peritoneal Cavity. J. Vis. Exp..

[30] Klymenko Y., Kim O., Loughran E., Yang J., Lombard R., Alber M., Stack M.S. (2017). Cadherin Composition and Multicellular Aggregate Invasion in Organotypic Models of Epithelial Ovarian Cancer Intraperitoneal Metastasis. Oncogene.

[31] Yin J.G., Liu X.Y., Wang B., Wang D.Y., Wei M., Fang H., Xiang M. (2016). Gene Expression Profiling Analysis of Ovarian Cancer. Oncol. Lett..

[32] Cretu A., Brooks P.C. (2007). Impact of the Non-Cellular Tumor Microenvironment on Metastasis: Potential Therapeutic and Imaging Opportunities. J. Cell. Physiol..

[33] Van Kempen L.C., Ruiter D.J., van Muijen G.N., Coussens L.M. (2003). The Tumor Microenvironment: A Critical Determinant of Neoplastic Evolution. Eur. J. Cell Biol..

[34] Ramaswamy S., Ross K.N., Lander E.S., Golub T.R. (2003). A Molecular Signature of Metastasis in Primary Solid Tumors. Nat. Genet..

[35] Xu L., Begum S., Hearn J.D., Hynes R.O. (2006). GPR56, an Atypical G Protein-Coupled Receptor, Binds Tissue Transglutaminase, TG2, and Inhibits Melanoma Tumor Growth and Metastasis. Proc. Natl. Acad. Sci. USA.

[36] Oudin M.J., Jonas O., Kosciuk T., Broye L.C., Guido B.C., Wyckoff J., Riquelme D., Lamar J.M., Asokan S.B., Whittaker C. (2016). Tumor Cell-Driven Extracellular Matrix Remodeling Drives Haptotaxis during Metastatic Progression. Cancer Discov..

[37] Gehler S., Ponik S.M., Riching K.M., Keely P.J. (2013). Bi-directional Signaling: Extracellular Matrix and Integrin Regulation of Breast Tumor Progression. Crit. Rev. Eukaryot. Gene Expr..

[38] Naba A., Clauser K.R., Hoersch S., Liu H., Carr S.A., Hynes R.O. (2012). The Matrisome: In Silico Definition and in vivo Characterization by Proteomics of Normal and Tumor Extracellular Matrices. Mol. Cell. Proteom..

[39] Cho A., Howell V.M., Colvin E.K. (2015). The Extracellular Matrix in Epithelial Ovarian Cancer—A Piece of a Puzzle. Front. Oncol..

[40] Paszek M.J., Zahir N., Johnson K.R., Lakins J.N., Rozenberg G.I., Gefen A., Reinhart-King C.A., Margulies S.S., Dembo M., Boettiger D. (2005). Tensional Homeostasis and the Malignant Phenotype. Cancer Cell.

[41] Butcher D.T., Alliston T., Weaver V.M. (2009). A Tense Situation: Forcing Tumour Progression. Nat. Rev. Cancer.

[42] Zhu G.G., Risteli L., Makinen M., Risteli J., Kauppila A., Stenback F. (1995). Immunohistochemical Study of Type I Collagen and Type I pN-Collagen in Benign and Malignant Ovarian Neoplasms. Cancer.

[43] Huijbers I.J., Iravani M., Popov S., Robertson D., Al-Sarraj S., Jones C., Isacke C.M. (2010). A Role for Fibrillar Collagen Deposition and the Collagen Internalization Receptor endo180 in Glioma Invasion. PLoS ONE.

[44] Kehlet S.N., Sanz-Pamplona R., Brix S., Leeming D.J., Karsdal M.A., Moreno V. (2016). Excessive Collagen Turnover Products are Released During Colorectal Cancer Progression and Elevated in Serum from Metastatic Colorectal Cancer Patients. Sci. Rep..

[45] Sherman-Baust C.A., Weeraratna A.T., Rangel L.B., Pizer E.S., Cho K.R., Schwartz D.R., Shock T., Morin P.J. (2003). Remodeling of the Extracellular Matrix through Overexpression of Collagen VI Contributes to Cisplatin Resistance in Ovarian Cancer Cells. Cancer Cell.

[46] Wu Y.H., Chang T.H., Huang Y.F., Huang H.D., Chou C.Y. (2014). COL11A1 Promotes Tumor Progression and Predicts Poor Clinical Outcome in Ovarian Cancer. Oncogene.

[47] Raglow Z., Thomas S.M. (2015). Tumor Matrix Protein Collagen XIalpha1 in Cancer. Cancer Lett..

[48] Teng P.N., Wang G., Hood B.L., Conrads K.A., Hamilton C.A., Maxwell G.L., Darcy K.M., Conrads T.P. (2014). Identification of Candidate Circulating Cisplatin-Resistant Biomarkers from Epithelial Ovarian Carcinoma Cell Secretomes. Br. J. Cancer.

[49] Pearce O.M.T., Delaine-Smith R.M., Maniati E., Nichols S., Wang J., Bohm S., Rajeeve V., Ullah D., Chakravarty P., Jones R.R. (2018). Deconstruction of a Metastatic Tumor Microenvironment Reveals a Common Matrix Response in Human Cancers. Cancer Discov..

[50] De Pascalis C., Etienne-Manneville S. (2017). Single and Collective Cell Migration: The Mechanics of Adhesions. Mol. Biol. Cell.

[51] Maziveyi M., Alahari S.K. (2017). Cell Matrix Adhesions in Cancer: The Proteins that Form the Glue. Oncotarget.

[52] Burridge K., Guilluy C. (2016). Focal Adhesions, Stress Fibers and Mechanical Tension. Exp. Cell Res..

[53] Tilghman R.W., Parsons J.T. (2008). Focal Adhesion Kinase as a Regulator of Cell Tension in the Progression of Cancer. Semin. Cancer Biol..

[54] Mitra A.K., Sawada K., Tiwari P., Mui K., Gwin K., Lengyel E. (2011). Ligand-Independent Activation of c-Met by Fibronectin and alpha(5)beta(1)-Integrin Regulates Ovarian Cancer Invasion and Metastasis. Oncogene.

[55] Kaur S., Kenny H.A., Jagadeeswaran S., Zillhardt M.R., Montag A.G., Kistner E., Yamada S.D., Mitra A.K., Lengyel E. (2009). β3-Integrin Expression on Tumor Cells Inhibits Tumor Progression, Reduces Metastasis, and is Associated with a Favorable Prognosis in Patients with Ovarian Cancer. Am. J. Pathol..

[56] Kobayashi M., Sawada K., Kimura T. (2017). Potential of Integrin Inhibitors for Treating Ovarian Cancer: A Literature Review. Cancers.

[57] Noh K., Mangala L.S., Han H.D., Zhang N., Pradeep S., Wu S.Y., Ma S., Mora E., Rupaimoole R., Jiang D. (2017). Differential Effects of EGFL6 on Tumor versus Wound Angiogenesis. Cell Rep..

[58] Choi H.J., Armaiz Pena G.N., Pradeep S., Cho M.S., Coleman R.L., Sood A.K. (2015). Anti-vascular Therapies in Ovarian Cancer: Moving beyond Anti-VEGF Approaches. Cancer Metastasis Rev..

[59] Lambert A.W., Pattabiraman D.R., Weinberg R.A. (2017). Emerging Biological Principles of Metastasis. Cell.

[60] Nakamura M., Ono Y.J., Kanemura M., Tanaka T., Hayashi M., Terai Y., Ohmichi M. (2015). Hepatocyte Growth Factor Secreted by Ovarian Cancer Cells Stimulates Peritoneal Implantation via the Mesothelial-Mesenchymal Transition of the Peritoneum. Gynecol. Oncol..

[61] Domcke S., Sinha R., Levine D.A., Sander C., Schultz N. (2013). Evaluating Cell Lines as Tumour Models by Comparison of Genomic Profiles. Nat. Commun..

[62] Haley J., Tomar S., Pulliam N., Xiong S., Perkins S.M., Karpf A.R., Mitra S., Nephew K.P., Mitra A.K. (2016). Functional Characterization of a Panel of High-Grade Serous Ovarian Cancer Cell Lines As Representative Experimental Models of the Disease. Oncotarget.

[63] Mitra A.K., Davis D.A., Tomar S., Roy L., Gurler H., Xie J., Lantvit D.D., Cardenas H., Fang F., Liu Y. (2015). In vivo Tumor Growth of high-grade Serous Ovarian Cancer Cell Lines. Gynecol. Oncol..

[64] Bolger A.M., Lohse M., Usadel B. (2014). Trimmomatic: A Flexible Trimmer for Illumina Sequence Data. Bioinformatics (Oxford, England).

[65] Kim D., Pertea G., Trapnell C., Pimentel H., Kelley R., Salzberg S.L. (2013). TopHat2: Accurate Alignment of Transcriptomes in the Presence of Insertions, Deletions and Gene Fusions. Genome Biol..

[66] Anders S., Pyl P.T., Huber W. (2015). HTSeq--a Python Framework to Work with High-Throughput Sequencing Data. Bioinformatics (Oxford, England).

[67] Love M.I., Huber W., Anders S. (2014). Moderated Estimation of Fold Change and Dispersion for RNA-seq Data with DESeq2. Genome Biol..

[68] Subramanian A., Tamayo P., Mootha V.K., Mukherjee S., Ebert B.L., Gillette M.A., Paulovich A., Pomeroy S.L., Golub T.R., Lander E.S. (2005). Gene Set Enrichment Analysis: A Knowledge-Based Approach for Interpreting Genome-Wide Expression Profiles. Proc. Natl. Acad. Sci. USA.

[69] Dorum G., Snipen L., Solheim M., Saebo S. (2009). Rotation Testing in Gene Set Enrichment Analysis for Small Direct Comparison Experiments. Stat. Appl. Genet. Mol. Biol..

