# Supplementary material for: Transcriptome Profiling Reveals Matrisome Alteration as a Key Feature of Ovarian Cancer Progression
Source: Cancers (Basel). 2019 Oct 9;11(10):1513. doi: 10.3390/cancers11101513 (PMC6826756; doi:10.3390/cancers11101513)
Supplement: Supplementary file 1 [file cancers-11-01513-s001.zip › Supplementary Figures S1.docx]

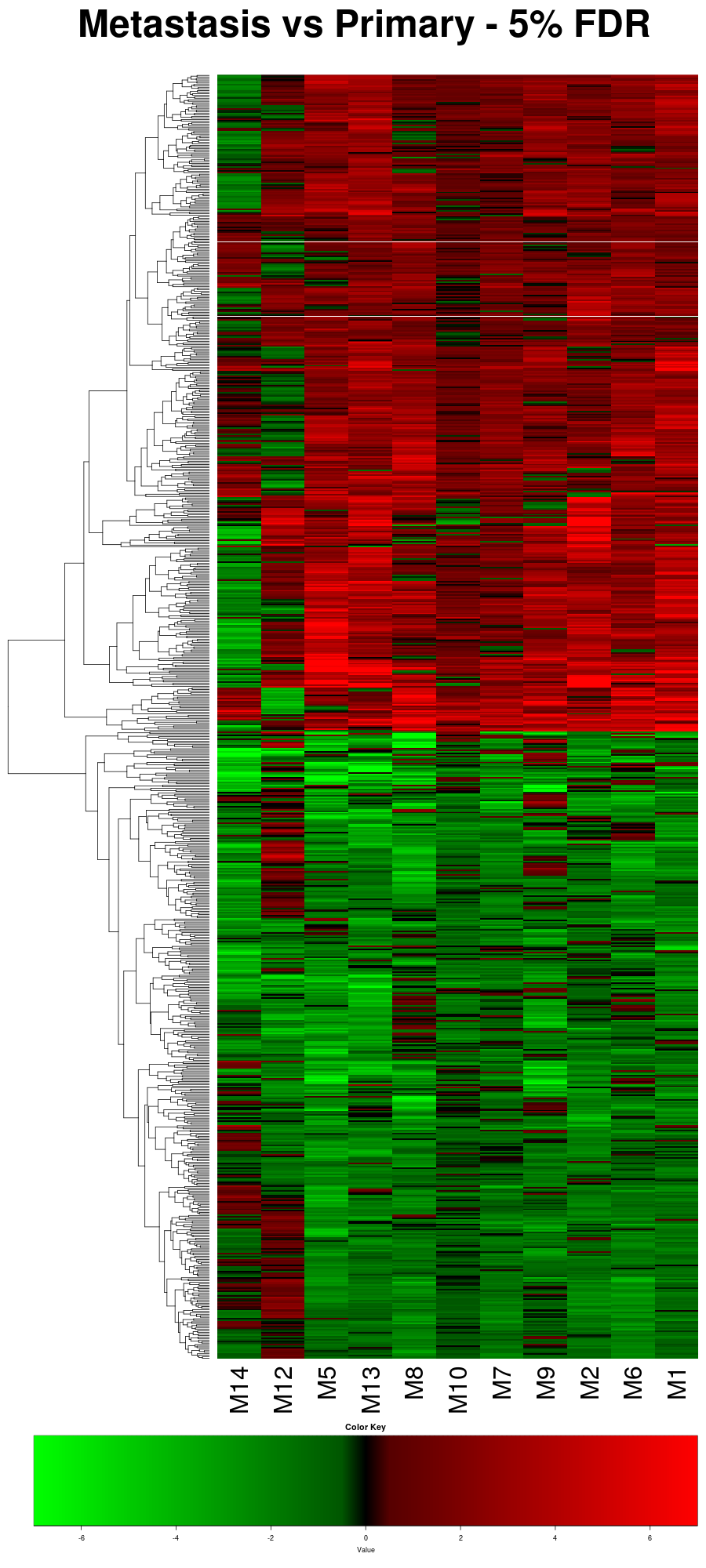


-6

-4

-2

0

2

4

6

**A**

**B**


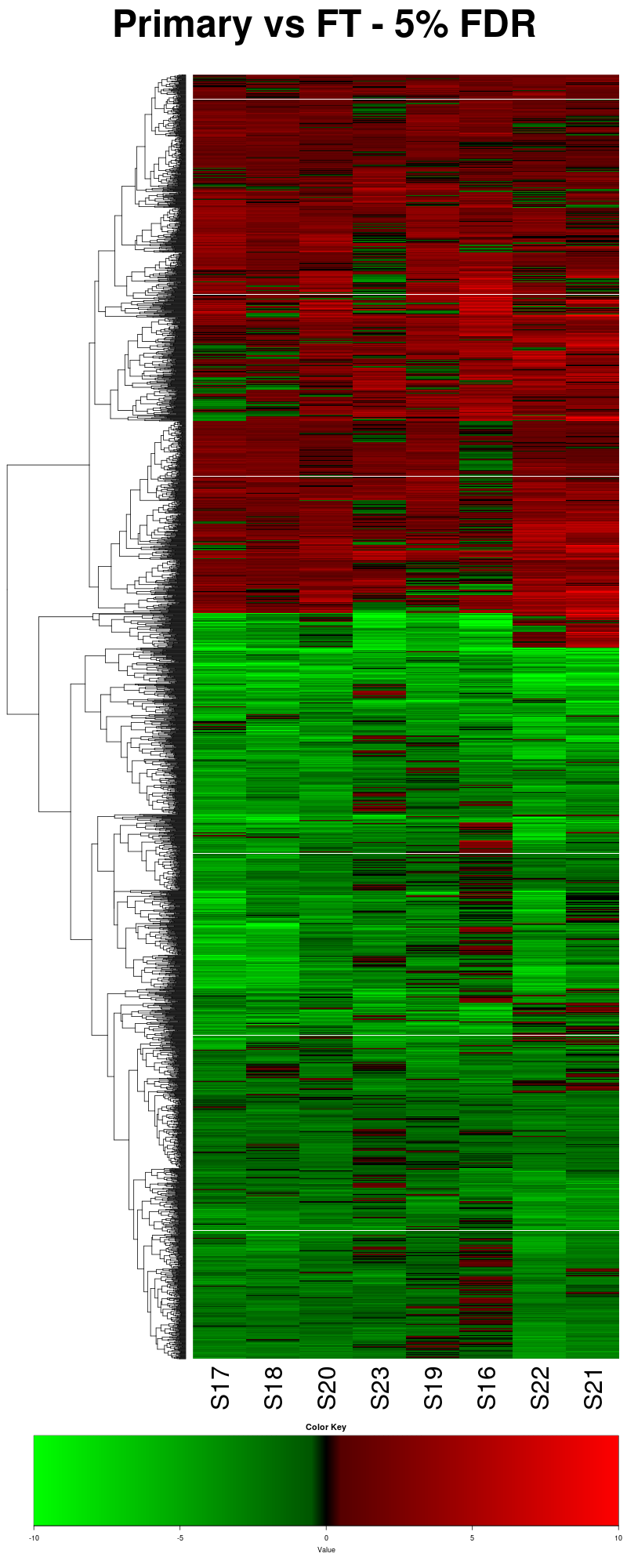


-10

-5

0

5

10

**Supplementary Figure S1:** Heat maps representing the deregulated genes in ovarian cancer **(A)** Primary tumor vs fallopian tube (FT) **(B)** Metastasis vs primary. Log2 ratio of normalized read counts from the significantly differentially expressed were hierarchically clustered and plotted into a heat map based on the log2 ratio values.
