# Supplementary material for: Transcriptome Profiling Reveals Matrisome Alteration as a Key Feature of Ovarian Cancer Progression
Source: Cancers (Basel). 2019 Oct 9;11(10):1513. doi: 10.3390/cancers11101513 (PMC6826756; doi:10.3390/cancers11101513)
Supplement: Supplementary file 1 [file cancers-11-01513-s001.zip › Supplementary Figures S2.docx]

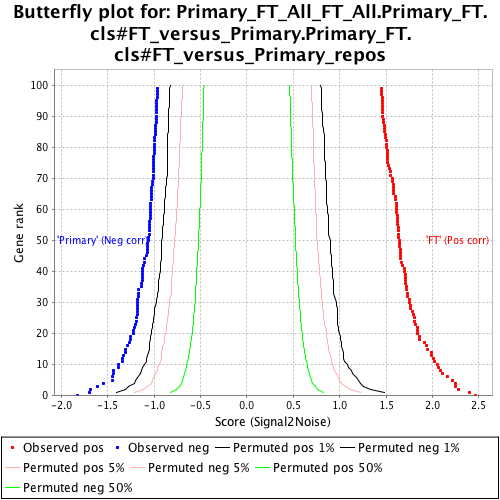

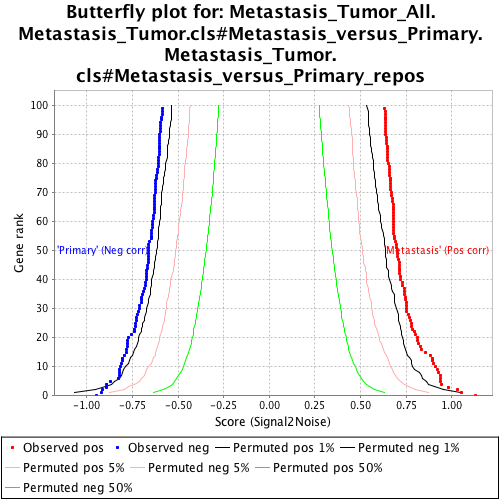


**Supplementary Figure S2:** Butterfly plots. **(A)** Butterfly plot representing the positive and negative correlation for top genes in primary tumors vs. FT. **(B)** Butterfly plot representing the positive and negative correlation for top genes in metastasis vs. primary tumors. Red and blue lines in the butterfly plots represent positive and negative correlation respectively while the black, pink and green lines in the butterfly plots depict permuted 1%, permuted 5% and permuted 50% respectively for both positive and negative correlation.

**A**

**B**
