# Supplementary material for: Transcriptome Profiling Reveals Matrisome Alteration as a Key Feature of Ovarian Cancer Progression
Source: Cancers (Basel). 2019 Oct 9;11(10):1513. doi: 10.3390/cancers11101513 (PMC6826756; doi:10.3390/cancers11101513)
Supplement: Supplementary file 1 [file cancers-11-01513-s001.zip › Supplementary Figures S3.docx]

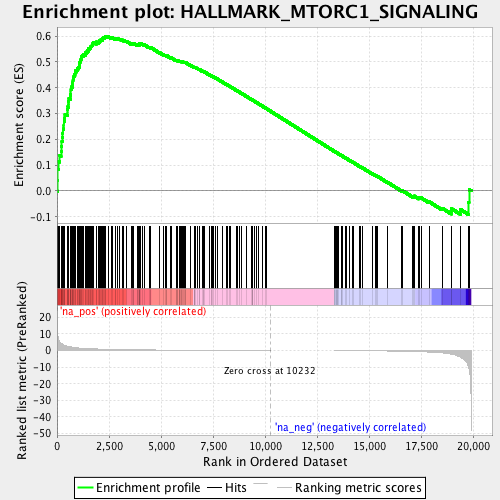

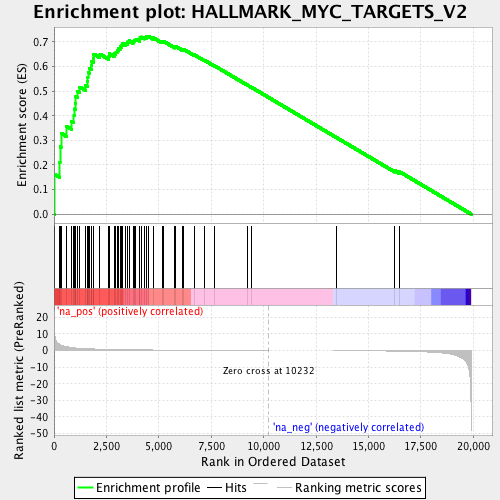

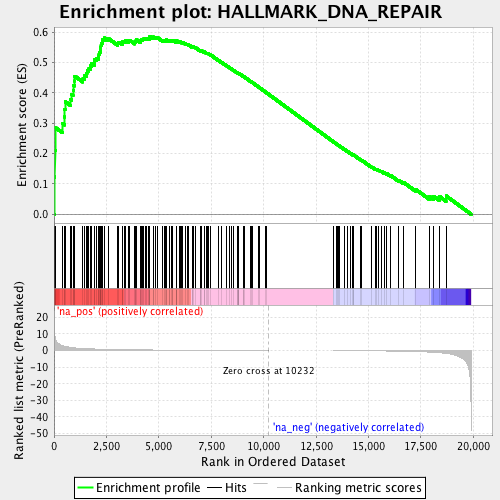

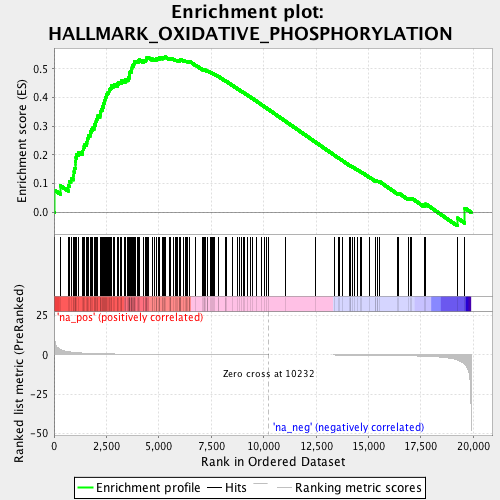

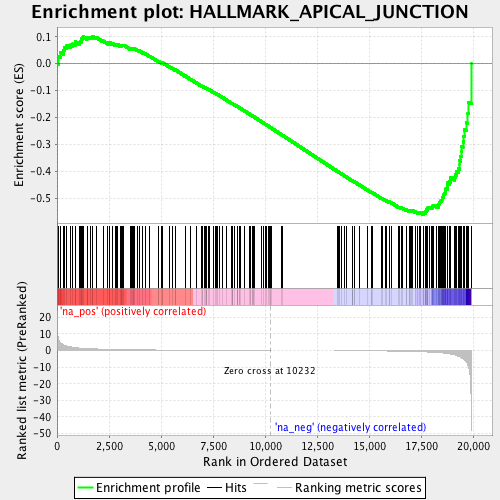

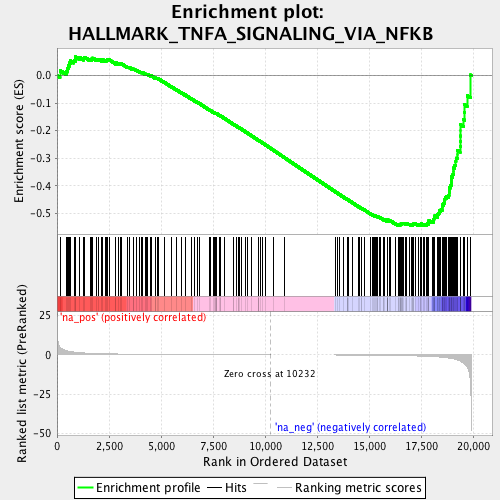

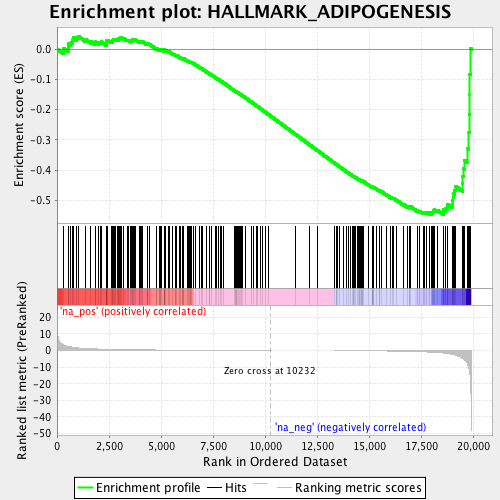

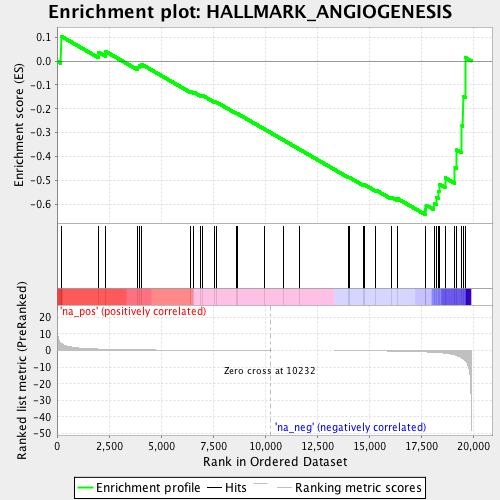


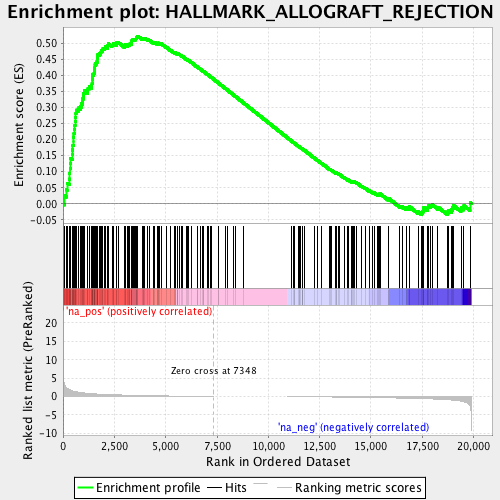

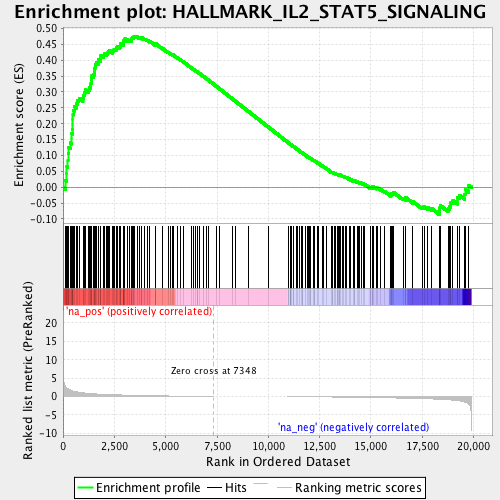

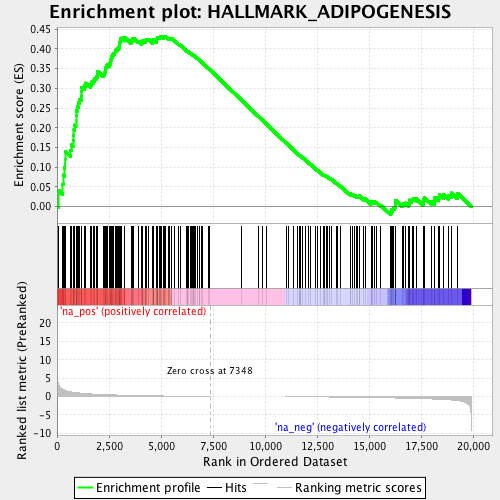

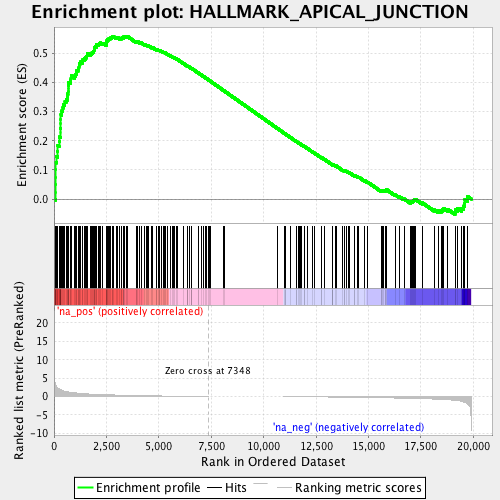

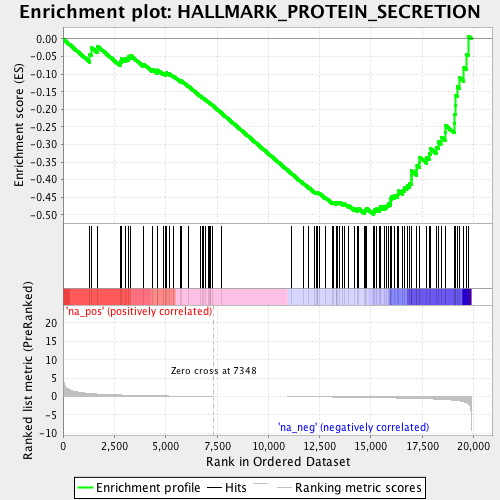

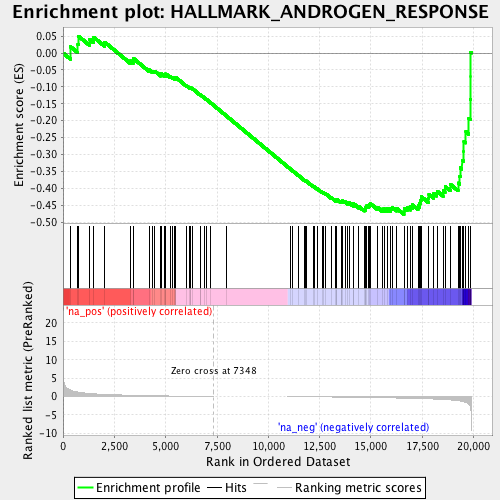

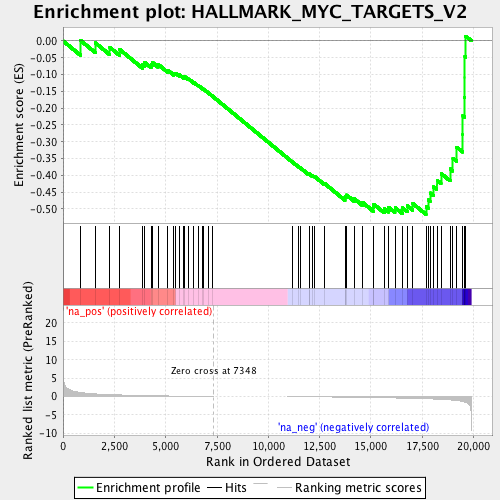

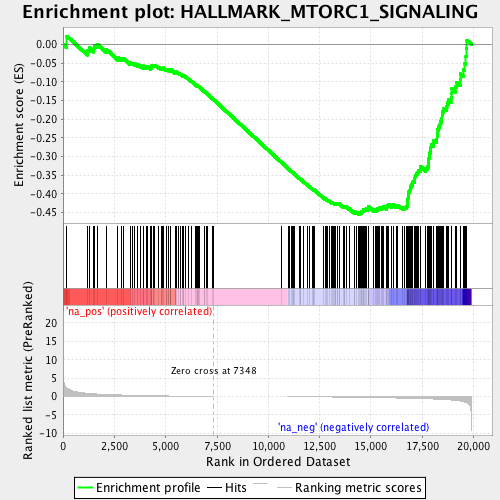


**Supplementary Figure S3:** Gene set enrichment analysis (GSEA) for the Hallmark gene set in ovarian cancer. **(A)** Primary tumor vs. FT (positively correlated). **(B)** Primary tumor vs. FT (negatively correlated). **(C)** Metastasis vs. primary tumors (positively correlated). **(D)** Metastasis vs. primary (negatively correlated).

**A**

**B**

**C**

**D**
