# Supplementary material for: Transcriptome Profiling Reveals Matrisome Alteration as a Key Feature of Ovarian Cancer Progression
Source: Cancers (Basel). 2019 Oct 9;11(10):1513. doi: 10.3390/cancers11101513 (PMC6826756; doi:10.3390/cancers11101513)
Supplement: Supplementary file 1 [file cancers-11-01513-s001.zip › Supplementary Figures S4-5.docx]

**A**

**B**

**Supplementary Figure S4:** Ingenuity pathway analysis (IPA) of differentially regulated genes in ovarian cancer. (**A**) Circular representation of overlapping canonical pathways for primary vs. fallopian tube. (**B**) Circular representation of overlapping canonical pathways for metastasis vs. primary.

**Supplementary Figure S5:** Heat map representing differentially expressed genes in all 3 HGSOC cells (Kuramochi/OVCAR4/OVCAR8) seeded on the 3D omentum culture vs. control. The color key represents the log2 fold change.


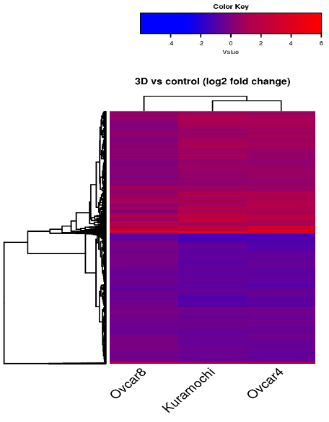

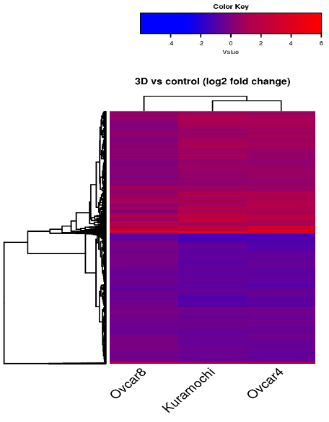


**3D omentum culture vs. control (log2 fold change)**

**OVCAR8**

**Kuramochi**

**OVCAR4**
