# Supplementary material for: Transcriptome Profiling Reveals Matrisome Alteration as a Key Feature of Ovarian Cancer Progression
Source: Cancers (Basel). 2019 Oct 9;11(10):1513. doi: 10.3390/cancers11101513 (PMC6826756; doi:10.3390/cancers11101513)
Supplement: Supplementary file 1 [file cancers-11-01513-s001.zip › Supplementary Table S1.docx]

**Supplementary Table S1: Ovarian cancer patient characteristics and specimen information.**

| **Metastasis vs. primary tumors** | | |  |  |  |  |  |
| --- | --- | --- | --- | --- | --- | --- | --- |
| **Code** | **Subject Age** | **Gender** | **Ethnicity** | **Race** | **Specific Site** | **Pathology Status** | **Histologic Type** |
| 1A | 61 | F | Non-Hispanic | White | OVARY | cancer-primary | SEROUS SURFACE PAPILLARY CARCINOMA |
| 1B | 61 | F | Non-Hispanic | White | PERITONEUM | cancer-metastasis | SEROUS SURFACE PAPILLARY CARCINOMA |
| 2A | 63 | F | Unknown | Unknown | OVARY | cancer-primary | PAPILLARY SEROUS ADENOCARCINOMA |
| 2B | 63 | F | Unknown | Unknown | OMENTUM | cancer-metastasis | PAPILLARY SEROUS ADENOCARCINOMA |
| 3A | 71 | F | Non-Hispanic | White | OVARY | cancer-primary | PAPILLARY SEROUS CARCINOMA |
| 3B | 71 | F | Non-Hispanic | White | PERITONEUM | cancer-metastasis | PAPILLARY SEROUS CARCINOMA |
| 4A | 42 | F | Non-Hispanic | White | OVARY | cancer-primary | PAPILLARY SEROUS CYSTADENOCARCINOMA |
| 4B | 42 | F | Non-Hispanic | White | PERITONEUM | cancer-metastasis | PAPILLARY SEROUS ADENOCARCINOMA |
| 5A | 76 | F | Non-Hispanic | White | OVARY | cancer-primary | PAPILLARY SEROUS ADENOCARCINOMA |
| 5B | 76 | F | Non-Hispanic | White | OMENTUM | cancer-metastasis | PAPILLARY SEROUS ADENOCARCINOMA |
| 6A | 55 | F | Non-Hispanic | White | OVARY | cancer-primary | PAPILLARY SEROUS CARCINOMA |
| 6B | 55 | F | Non-Hispanic | White | OMENTUM | cancer-metastasis | PAPILLARY SEROUS CARCINOMA |
| 7A | 47 | F | Non-Hispanic | White | OVARY | cancer-primary | PAPILLARY SEROUS CARCINOMA |
| 7B | 47 | F | Non-Hispanic | White | OMENTUM | cancer-metastasis | PAPILLARY SEROUS CARCINOMA |
| 8A | 70 | F | Non-Hispanic | White | OVARY | cancer-primary | SEROUS ADENOCARCINOMA |
| 8B | 70 | F | Non-Hispanic | White | OMENTUM | cancer-metastasis | SEROUS ADENOCARCINOMA |
| 9A | 65 | F | Non-Hispanic | White | OVARY | cancer-primary | SEROUS CARCINOMA |
| 9B | 65 | F | Non-Hispanic | White | OMENTUM | cancer-metastasis | SEROUS CARCINOMA |
| 10A | 62 | F | Non-Hispanic | White | OVARY | cancer-primary | PAPILLARY SEROUS CARCINOMA |
| 10B | 62 | F | Non-Hispanic | White | OMENTUM | cancer-metastasis | PAPILLARY SEROUS CARCINOMA |
| 11A | 49 | F | Non-Hispanic | White | OVARY | cancer-primary | PAPILLARY SEROUS ADENOCARCINOMA |
| 11B | 49 | F | Non-Hispanic | White | OMENTUM | cancer-metastasis | PAPILLARY SEROUS ADENOCARCINOMA |
| 12A | 84 | F | Non-Hispanic | White | OVARY | cancer-primary | PAPILLARY SEROUS CYSTADENOCARCINOMA |
| 12B | 84 | F | Non-Hispanic | White | OMENTUM | cancer-metastasis | PAPILLARY SEROUS CYSTADENOCARCINOMA |
| 13A | 50 | F | Non-Hispanic | White | OVARY | cancer-primary | PAPILLARY SEROUS CYSTADENOCARCINOMA |
| 13B | 50 | F | Non-Hispanic | White | OMENTUM | cancer-metastasis | PAPILLARY SEROUS CYSTADENOCARCINOMA |
| 14A | 78 | F | Non-Hispanic | White | OVARY | cancer-primary | PAPILLARY SEROUS CARCINOMA |
| 14B | 78 | F | Non-Hispanic | White | OMENTUM | cancer-metastasis | PAPILLARY SEROUS CARCINOMA |
| **Primary vs. FT** | |  |  |  |  |  |  |
| **Code** | **Subject Age** | **Gender** | **Ethnicity** | **Race** | **Specific Site** | **Pathology Status** | **Histologic Type** |
| 15FT | 46 | F | Non-Hispanic | White | FALLOPIAN TUBE | normal adjacent | NORMAL TISSUE |
| 15P | 46 | F | Non-Hispanic | White | OVARY | cancer-primary | ENDOMETRIOID ADENOCARCINOMA |
| 16FT | 52 | F | Non-Hispanic | White | FALLOPIAN TUBE | normal adjacent | NORMAL TISSUE |
| 16P | 52 | F | Non-Hispanic | White | OVARY | cancer-primary | CLEAR CELL ADENOCARCINOMA, NOS |
| 17FT | 51 | F | Non-Hispanic | White | FALLOPIAN TUBE | normal adjacent | NORMAL TISSUE |
| 17P | 51 | F | Non-Hispanic | White | OVARY | cancer-primary | MIXED CLEAR CELL, ENDOMETRIOID AND PAPILLARY SEROUS CARCINOMAS |
| 18FT | 46 | F | Non-Hispanic | White | FALLOPIAN TUBE | normal adjacent | NORMAL TISSUE |
| 18P | 46 | F | Non-Hispanic | White | OVARY | cancer-primary | PAPILLARY SEROUS ADENOCARCINOMA |
| 19FT | 47 | F | Non-Hispanic | White | FALLOPIAN TUBE | normal adjacent | NORMAL TISSUE |
| 19P | 47 | F | Non-Hispanic | White | OVARY | cancer-primary | ENDOMETRIOID ADENOCARCINOMA |
| 20FT | 58 | F | Non-Hispanic | White | FALLOPIAN TUBE | normal adjacent | NORMAL TISSUE |
| 20P | 58 | F | Non-Hispanic | White | OVARY | cancer-primary | SEROUS CARCINOMA |
| 21FT | 45 | F | Non-Hispanic | White | FALLOPIAN TUBE | normal adjacent | NORMAL TISSUE |
| 21P | 45 | F | Non-Hispanic | White | OVARY | cancer-primary | SEROUS CARCINOMA |
| 22FT | 58 | F | Non-Hispanic | White | FALLOPIAN TUBE | normal adjacent | NORMAL TISSUE |
| 22P | 58 | F | Non-Hispanic | White | OVARY | cancer-primary | PAPILLARY SEROUS AND ENDOMETRIOID ADENOCARCINOMA |
| 23FT | 45 | F | Non-Hispanic | White | FALLOPIAN TUBE | normal adjacent | NORMAL TISSUE |
| 23P | 45 | F | Non-Hispanic | White | OVARY | cancer-primary | SEROUS SURFACE PAPILLARY CARCINOMA |
