# Supplementary material for: Transcriptome Profiling Reveals Matrisome Alteration as a Key Feature of Ovarian Cancer Progression
Source: Cancers (Basel). 2019 Oct 9;11(10):1513. doi: 10.3390/cancers11101513 (PMC6826756; doi:10.3390/cancers11101513)
Supplement: Supplementary file 1 [file cancers-11-01513-s001.zip › Supplementary Table S7.docx]

**Supplementary Table S7: KM plotter settings for progression free survival analysis (PFS).**

| **Gene** | **Affy ID** | **Split by** | **Censure at threshold** | **Histology/**  **Stage/Grade** | **TP53 mutation** | **Debulk/**  **Chemotherapy** | **Survival** |
| --- | --- | --- | --- | --- | --- | --- | --- |
| EPYC | 206439_at | Median | Yes | All | All | All patients | PFS |
| COL10A1 | 217428_s_at | Median | Yes | All | All | All patients | PFS |
| PLPP4 | 236044_at | Median | Yes | All | All | All patients | PFS |
| AGR3 | 228241_at | Median | Yes | All | All | All patients | PFS |
| SNTN | 239150_at | Median | Yes | All | All | All patients | PFS |
| COL28A1 | 242345_at | Median | Yes | All | All | All patients | PFS |
| PRSS16 | 208165_s_at | Median | Yes | All | All | All patients | PFS |
| STAR | 204548_at | Median | Yes | All | All | All patients | PFS |
| NR0B1 | 206645_s_at | Median | Yes | All | All | All patients | PFS |
| MEIS3 | 228327_x_at | Median | Yes | All | All | All patients | PFS |
| ITGA11 | 222899_at | Median | Yes | All | All | All patients | PFS |
| LYL1 | 210044_s_at | Median | Yes | All | All | All patients | PFS |
