# Supplementary material for: Transcriptome Profiling Reveals Matrisome Alteration as a Key Feature of Ovarian Cancer Progression
Source: Cancers (Basel). 2019 Oct 9;11(10):1513. doi: 10.3390/cancers11101513 (PMC6826756; doi:10.3390/cancers11101513)
Supplement: Supplementary file 1 [file cancers-11-01513-s001.zip › Supplementary Table S6.doc]

**Supplementary Table S6: Common genes in metastasis vs.** primary tumors and primary tumors vs. FT.

| **(A). Common upregulated genes in ovarian cancer metastasis vs. primary and primary vs. FT** | | | **(B). Common downregulated genes in ovarian cancer metastasis vs. primary and primary vs. FT** | | |
| --- | --- | --- | --- | --- | --- |
| **Gene Name** | **log2 fold change** | ***p*-value** | **Gene Name** | **log2 fold change** | ***p*-value** |
| EPYC | 4.84 | 2.78 × 10^−28^ | AGR3 | −2.09 | 2.25 × 10^−10^ |
| COL10A1 | 1.98 | 5.19 × 10^−7^ | SNTN | −3.09 | 4.80 × 10^−5^ |
| PLPP4 | 2.06 | 5.95 × 10^−5^ | ANKUB1 | −3.41 | 1.64 × 10^−5^ |
| RP11**-**13P5.2 | 2.44 | 2.46 × 10^−5^ | COL28A1 | −1.29 | 0.000236 |
| PODNL1 | 1.44 | 0.002166 | DCDC2B | −3.01 | 0.001219 |
| CILP2 | 1.54 | 0.000325 | ADGB | −2.75 | 0.000667 |
| FNDC1 | 2.32 | 3.26 × 10^−5^ | CFAP52 | −1.88 | 1.19 × 10^−5^ |
| SLC35D3 | 2.09 | 0.000901 | RP11−356K23.1 | −2.77 | 0.001993 |
|  |  |  | WDR38 | −1.49 | 8.60 × 10^−5^ |
|  |  |  | CFAP221 | −1.19 | 0.000604 |
|  |  |  | NWD1 | −1.72 | 0.000766 |
|  |  |  | FAM166B | −1.07 | 0.001892 |
| **(C). Top 10 opposite: Upregulated genes in metastasis vs. primary and downregulated in primary vs. FT** | | | **(D). Top 10 opposite: Downregulated genes in metastasis vs. primary and upregulated in primary vs. FT** | | |
| **Gene Name** | **log2 fold change** | ***p*-value** | **Gene Name** | **log2 fold change** | ***p*-value** |
| MEIS3 | 2.04 | 1.29 × 10^−14^ | ATP6V1C2 | −1.73 | 6.83 × 10^−7^ |
| ITGA11 | 2.19 | 1.58 × 10^−7^ | STAR | −1.67 | 4.38 × 10^−8^ |
| RP1**-**79C4.4 | 1.10 | 2.54E × 10^−8^ | NR0B1 | −1.84 | 5.01 × 10^−4^ |
| LYL1 | 1.42 | 9.63 × 10^−7^ | KCNG3 | −1.58 | 9.16 × 10^−4^ |
| ASPA | 1.49 | 3.70 × 10^−5^ | PRSS16 | −1.14 | 3.02 × 10^−5^ |
| AOC3 | 1.49 | 9.69 × 10^−6^ | FAM167A | −1.45 | 6.31 × 10^−5^ |
| GIMAP8 | 1.41 | 4.79 × 10^−8^ | C3orf67 | −1.30 | 1.00 × 10^−4^ |
| LRRN4CL | 1.23 | 5.73 × 10^−5^ | JPH1 | −1.62 | 1.85 × 10^−4^ |
| IGHV1**-**24 | 1.99 | 5.70 × 10^−4^ | HOOK1 | −1.56 | 1.93 × 10^−5^ |
| CCL14 | 2.85 | 7.45 × 10^−6^ | ESM1 | −1.78 | 5.89 × 10^−5^ |
